# VARGG: a deep learning framework advancing precise spatial domain identification and cellular heterogeneity analysis in spatial transcriptomics

**DOI:** 10.1093/bfgp/elaf018

**Published:** 2025-11-23

**Authors:** Mengqiu Wang, Zhiwei Zhang, Lixin Lei, Kaitai Han, Zhenghui Wang, Ruoyan Dai, Zijun Wang, Chaojing Shi, Xudong Zhao, Qianjin Guo

**Affiliations:** Academy of Artificial Intelligence, Beijing Institute of Petrochemical Technology, 19 Qingyuan North Road, Daxing District, Beijing 102617, China; Academy of Artificial Intelligence, Beijing Institute of Petrochemical Technology, 19 Qingyuan North Road, Daxing District, Beijing 102617, China; Academy of Artificial Intelligence, Beijing Institute of Petrochemical Technology, 19 Qingyuan North Road, Daxing District, Beijing 102617, China; Academy of Artificial Intelligence, Beijing Institute of Petrochemical Technology, 19 Qingyuan North Road, Daxing District, Beijing 102617, China; Academy of Artificial Intelligence, Beijing Institute of Petrochemical Technology, 19 Qingyuan North Road, Daxing District, Beijing 102617, China; Academy of Artificial Intelligence, Beijing Institute of Petrochemical Technology, 19 Qingyuan North Road, Daxing District, Beijing 102617, China; Academy of Artificial Intelligence, Beijing Institute of Petrochemical Technology, 19 Qingyuan North Road, Daxing District, Beijing 102617, China; Academy of Artificial Intelligence, Beijing Institute of Petrochemical Technology, 19 Qingyuan North Road, Daxing District, Beijing 102617, China; Academy of Artificial Intelligence, Beijing Institute of Petrochemical Technology, 19 Qingyuan North Road, Daxing District, Beijing 102617, China; Academy of Artificial Intelligence, Beijing Institute of Petrochemical Technology, 19 Qingyuan North Road, Daxing District, Beijing 102617, China

**Keywords:** spatial transcriptomics, variational graph autoencoder, multi-head attention, spatial clustering, deep learning

## Abstract

Spatial transcriptomics has revolutionized our ability to measure gene expression while preserving spatial information, thus facilitating detailed analysis of tissue structure and function. Identifying spatial domains accurately is key for understanding tissue microenvironments and biological progression. To overcome the challenge of integrating gene expression data with spatial information, we introduce the VARGG deep learning framework. VARGG combines a pretrained Vision Transformer (ViT) with a graph neural network autoencoder, utilizing ViT’s self-attention mechanism to capture global contextual information and enhance understanding of spatial relationships. This framework is further enhanced by multi-layer gated residual graph neural networks and Gaussian noise, which improve feature representation and model generalizability across different data sources. The robustness and scalability of VARGG have been verified on different platforms (10x Visium, Slide-seqV2, Stereo-seq, and MERFISH) and datasets of different sizes (human glioblastoma, mouse embryo, breast cancer). Our results demonstrate that VARGG’s ability to accurately delineate spatial domains can provide a deeper understanding of tissue structure and help identify key molecular markers and potential therapeutic targets, thereby improving our understanding of disease mechanisms and providing opportunities for personalization to inform the development of treatment strategies.

## Introduction

In spatial transcriptomics (ST) [1], identifying spatial domains is crucial for understanding cellular heterogeneity [2] and the dynamic changes in tissue microenvironments. Spatial domains are defined as continuous tissue regions with consistent gene expression and histological characteristics, with significant value in clinical applications such as disease subtyping, prognosis assessment, and therapeutic target discovery.

Current methods for identifying spatial domains are categorized into non-spatial and spatial clustering algorithms. Non-spatial clustering algorithms (such as Seurat [3] and SCANPY [4]) cluster data based on gene expression similarity without considering spatial context, often generating biological patterns inconsistent with actual tissue structures due to ignoring spatial background information. Spatial clustering algorithms, on the other hand, consider spatial information but also have their limitations. Giotto [5] implements data integration by constructing customizable spatial neighborhood networks, but has limited fine-grained detection capabilities; BayesSpace [6] employs Bayesian clustering to enhance resolution, but requires pre-specified cluster numbers. Deep learning methods have been widely applied in this field, but these methods still have some limitations. SpaGCN [7] integrates gene expression data with spatial neighborhood information for clustering, but has limited efficiency when analyzing complex heterogeneous tissues; DeepST [8] utilizes deep learning techniques to extract complex features from high-dimensional data, but requires substantial computational resources and faces challenges in multimodal integration; stLearn [9] utilizes transfer learning to integrate histological images, but relies on heuristic spatial smoothing strategies; SEDR [10] focuses on non-linear dimensionality reduction, but has limited capability in integrating tissue morphological information; STAGATE [11] preserves spatial topological relationships through graph attention autoencoders, but is constrained by predefined neighborhood selection parameters; GraphST [12] combines graph neural networks with self-supervised contrastive learning to optimize clustering accuracy, but shows unstable performance with highly heterogeneous data and may lose critical data features when using decoder output as latent space; SpaCAE [13] uses graph embedding variational autoencoders [14] and deep contrastive strategies to detect fine-grained tissue structures, but faces computational complexity and parameter tuning challenges in handling spatial relationships and integrating multidimensional information.

Recently, two breakthrough computational frameworks have emerged in the field of spatial domain discovery. CellTransformer [15], based on transformer architecture, is specifically designed for spatial transcriptomics analysis of the mouse brain, capable of identifying spatial domains from coarse to fine granularity while maintaining consistency with the Allen brain atlas. NicheCompass [16] utilizes a graph deep learning approach based on cell communication principles, deciphering microenvironments by integrating knowledge of intercellular interactions, demonstrating excellent performance in the analysis of various tissue samples, including successful application to a spatial atlas of the mouse brain comprising 8.4 million cells.

These powerful frameworks represent significant progress, yet the field continues to rapidly evolve in pursuit of ever-greater technical refinement. As highlighted in recent reviews [17], researchers are actively addressing persistent challenges such as batch effect correction, dynamic fusion of multimodal data, and sophisticated spatial neighborhood construction. In this vein, emerging methods like SPACEL [18] and GRAS4T [19] have proposed novel strategies to tackle some of these technical aspects. However, this intense focus on creating a universally superior algorithm can overshadow the need for tools specifically optimized for high-impact biological questions. General-purpose tools, while versatile, may not provide the required analytical depth for hypothesis-driven research in complex diseases. For instance, studying the nuanced molecular and morphological changes within key hippocampal subregions (e.g. CA1, CA3, DG)—critical areas affected in early-stage Alzheimer’s disease (AD)—requires a focused approach that can capture subtle pathological signatures. This highlights a gap for methods designed not merely for universal applicability, but for high-precision analysis within specific and clinically significant contexts.

To address this gap, we propose VARGG, a new method designed to provide a high-precision spatial transcriptomics analysis solution for studying complex tissue architectures in disease contexts. Its architecture employs a pretrained Vision Transformer (ViT) [20] and self-attention [21] mechanisms to effectively capture the global context from histology images, enabling the identification of subtle morphological alterations often associated with neurodegeneration. By integrating morphological information from ViT with spatial location and gene expression data, VARGG generates enhanced latent representations through a multi-layer gated residual network, improving its ability to learn complex features in biological tissues. Furthermore, the model incorporates multi-head attention and Gaussian noise [22] handling to enhance robustness against data variability and noise. This design results in the precise identification of spatial domains, proving particularly effective in delineating hippocampal subregions and their molecular characteristics relevant to AD. We validated VARGG’s performance and generalizability across diverse platforms, including 10x Visium, Slide-seqV2 [23], Stereo-seq [24], and MERFISH [25], demonstrating its capability to handle large-scale spatial omics data in different biological contexts. In summary, VARGG provides an efficient, high-precision method for spatial domain mapping that serves as a complementary framework to general-purpose tools like CellTransformer and NicheCompass, showcasing the value of tailoring computational models to specific biological contexts to drive hypothesis-driven discovery.

## Results

### The overall architecture of VARGG

**Figure 1:**
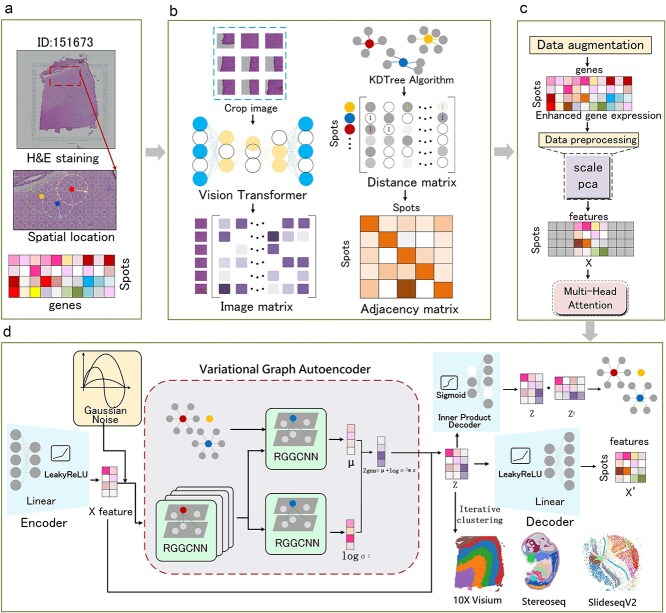
VARGG model for spatial transcriptomics analysis. (**a**) Overview of the VARGG model integrating tissue images, spatial coordinates, and gene expression data. (**b**) Left: extraction of morphological features from H&E images using ViT; right: construction of adjacency matrix by calculating spatial distances using KDTree. (**c**) Data augmentation: enhancement of feature matrix by combining morphological features, spatial information, and gene expression data through multi-head attention mechanisms. (**d**) Variational autoencoder with RGGCNN to generate latent variable *Z*, followed by Leiden clustering to identify spatial domains.

We introduce a novel VARGG model that integrates tissue slice images, spatial location information, and gene expression data (Fig. 1a). The model employs a pretrained ViT to process H&E-stained image blocks, capturing intricate features using an attention mechanism that considers positional information and focuses on multiple subspaces. Layer normalization stabilizes training and accelerates convergence. KDTree calculates a distance matrix between spots, converted into an adjacency matrix representing spot connectivity (Fig. 1b). Combining these matrices with gene expression data creates a feature matrix, processed by a multi-head attention mechanism to capture complex spatial patterns and gene expression relationships (Fig. 1c). This matrix then enters a denoising encoder for deeper feature learning and clustering analysis, with Gaussian noise added to prevent overfitting. The variational autoencoder uses multi-layer ResGatedGraphConv Network (RGGCNN) [26] to capture intricate spatial relationships, learning key parameters (mean and log variance) to define a latent space for generating the latent variable *Z*. The decoder reconstructs the data, optimizing the feature matrix to reveal deeper structures. Finally, the Leiden method clusters *Z* to identify spatial domains (Fig. 1d).

### VARGG demonstrates superior accuracy in spatial domain recognition in the human dorsolateral prefrontal cortex ST dataset compared to existing models

**Figure 2:**
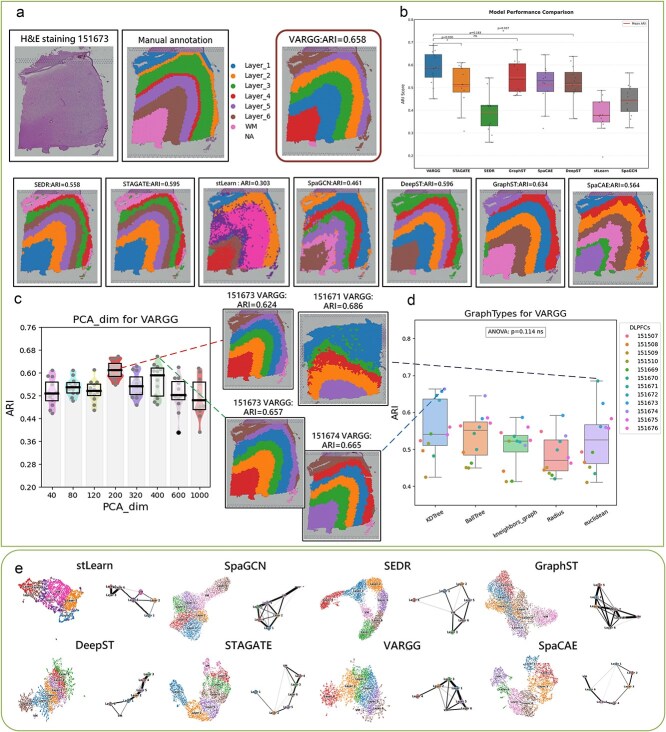
Performance of VARGG on human dorsolateral prefrontal cortex (DLPFC) spatial transcriptomics dataset and comparison with other methods. (**a**) Left side shows the H&E-stained image of the human dorsolateral prefrontal cortex (DLPFC) from the original study and its corresponding manual annotation, while the bottom displays a comparison of clustering results by eight methods, VARGG, SEDR, STAGATE, stLearn, SpaGCN, DeepST, GraphST, and SpaCAE, on slice 151673 of the DLPFC dataset. (**b**) Boxplot comparison of Adjusted Rand Index (ARI) scores of 8 methods across 12 DLPFC slices. (**c**) VARGG performance across different PCA dimensions. (**d**) ARI scores of VARGG using five different adjacency matrix construction methods. (**e**) UMAP and PAGA visualization comparison of 8 models.

VARGG demonstrates excellent accuracy in spatial domain identification on the dorsolateral prefrontal cortex (DLPFC) [27] dataset. In this experiment, we employed eight cutting-edge models to identify spatial domains in the H&E-stained tissue section (ID: 151673). Maynard *et al.* manually annotated the cortical layers (L1–L6) and white matter of the DLPFC slides by gene marker and cytoarchitecture as shown in Fig. 2a. We calculated the Adjusted Rand Index (ARI) [28] scores to assess the accuracy of each model, where values closer to 1 indicate higher consistency with the true labels. Among all the models tested, VARGG achieved the highest ARI score of 0.658 on sample 151673. As shown in Fig. 2a, a visual comparison reveals that stLearn and SpaGCN performed the worst, failing to recover the first-layer cluster, with the remaining clusters mixed. SEDR, STAGATE, SpaCAE, and DeepST could recover most layers, but with irregular boundaries. In contrast, GraphST and VARGG generated layers with shapes very similar to the annotations. The domains identified by VARGG exhibited smoother boundaries and less noise, achieving a higher ARI score.

To verify the stability of VARGG and eliminate experimental randomness, we conducted tests on 12 slices from the DLPFC dataset. The visualization results of these slices are shown in Fig. 2a and Fig. S1, while Fig. 2b presents the quantitative analysis results. VARGG achieved the highest median ARI score of 0.586 and statistically outperformed STAGATE (*P* = .030^*^) and DeepST (*P* = .037^*^). VARGG’s median ARI score was numerically higher than GraphST, but statistical analysis (*P* = .183 ns) indicated no significant difference between the two. Other methods such as SEDR, SpaCAE, stLearn, and SpaGCN had median ARI scores <0.54. The statistical results demonstrate that VARGG exhibits stronger performance advantages in spatial transcriptomics analysis tasks compared to most benchmark methods.

We also evaluated the impact of different adjacency matrix construction methods and dimensionality reduction on VARGG’s performance using the DLPFC dataset. First, we analyzed the effect of dimensionality reduction on VARGG. Figure 2c displays a box plot showing the distribution of ARI values across different PCA dimensions for VARGG. Each box represents multiple data points for the corresponding dimension while also displaying the median. The results indicate that VARGG maintains stable performance within the 80–320 dimension range. Next, we compared the effectiveness of different adjacency matrix construction methods. Figure 2d presents box plots showing the distribution of ARI values for each method across 12 slices from the DLPFC dataset. Despite differences in construction processes, their ARI values did not show significant differences. To statistically analyze these differences, we employed one-way ANOVA. The ANOVA results (*F* = 1.9591, *P* = .1136) indicate no statistically significant differences in ARI values among the different graph construction methods. Our analysis demonstrates that the VARGG algorithm excels in preserving complex intercellular relationships while maintaining cellular population structures, which is crucial for understanding developmental trajectories and functional interactions. As shown in Fig. 2e, compared to other algorithms, VARGG can simultaneously display clear separation of cell populations and meaningful connections between groups. In contrast, methods such as stLearn and SpaGCN, while capable of identifying major cell populations, often simplify or lose critical information about intercellular associations, potentially leading to biases in understanding tissue functional organization.

In terms of computational performance, VARGG processes data containing 4000 spots and 30 000 genes in ~6.5 min (running on GPU), which is slightly better than DeepST but somewhat slower than lightweight models like GraphST. VARGG’s memory requirement is ~6 GB, primarily due to its need to simultaneously process spatial transcriptomics data and construct complex graph structures. Nevertheless, considering the high accuracy of spatial domain identification that VARGG provides, this computational resource consumption remains within a reasonable range. Detailed comparisons of memory usage and runtime across models are provided in Fig. S2. This balanced computational efficiency makes VARGG a practical choice for analyzing large-scale spatial transcriptomics data, especially when dealing with complex tissues like the DLPFC, providing high-quality analysis results within reasonable computational resource constraints.

Overall, VARGG demonstrates effective partitioning of spatial domains in complex brain regions, improved identification of cellular heterogeneity, and enhanced modeling of cell interactions. These capabilities position VARGG as a valuable tool for spatial transcriptomics analysis. In neuroscience and cancer research contexts, VARGG’s computational advantages contribute to better characterization of tissue architecture and cellular relationships, enabling researchers to explore spatial gene expression patterns in disease-relevant tissues with increased precision.

### Identification of key therapeutic target genes for glioblastoma by VARGG model analysis

**Figure 3:**
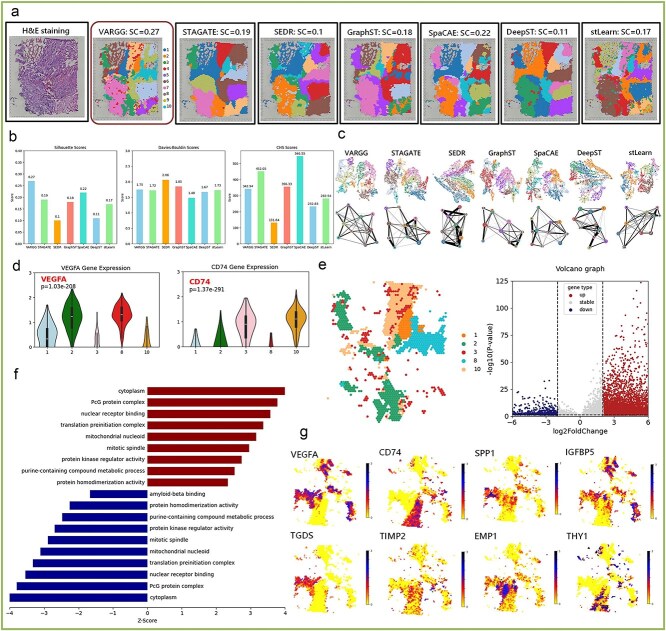
Human glioblastoma spatial transcriptomics clustering and gene expression analysis assessment: (**a**) comparison of spatial clustering algorithms on glioblastoma tissue. H&E staining (left) and resulting clusters from different methods with their respective silhouette coefficient (SC) values. (**b**) Performance metrics comparison of clustering algorithms. Bar charts show silhouette scores (left), Davies–Bouldin scores (middle), and Calinski–Harabasz scores (right) across methods. (**c**) Comparison of UMAP visualizations (top) and PAGA trajectory graphs (bottom) across different algorithms. VARGG maintains both clear cluster boundaries and meaningful inter-cluster connections compared to other methods. (**d**) VEGFA and CD74 expression across cell clusters [1, 3, 8, 10]. VEGFA is significantly elevated in cluster 2 (*P* = 1.03e−208); CD74 shows the highest expression in clusters 10 (*P* = 1.37e−291). (**e**) Left: UMAP visualization of five identified clusters in spatial transcriptomic data. Right: Volcano plot visualizing differential gene expression, showing significantly differentially expressed genes in domains 1, 2, 3, 8, 10. (**f**) Bar chart of Gene Ontology (GO) enrichment analysis showing enriched biological processes and molecular functions’ *Z*-scores, indicating overexpressed pathways in identified clusters. (**g**) Spatial expression plots of selected genes showing their expression distribution in glioblastoma tissue sections.

In our experiments, we evaluated seven spatial transcriptomic algorithms on the human glioblastoma [29] dataset from the 10X platform (Fig. 3a). VARGG achieved the highest silhouette coefficient (SC = 0.27) among all methods, demonstrating its superior ability to generate well-separated cell clusters with biological significance. As shown in Fig. 3b, VARGG maintained balanced performance across multiple clustering quality metrics. While not leading in all measures, it ranked competitively in the Davies–Bouldin index (DBI) [30] and Calinski–Harabasz (CH) [31] score, outperforming most competing methods. These complementary metrics collectively validate VARGG’s accuracy and stability in capturing cell population distributions within the complex glioblastoma microenvironment. The comparison of data representation approaches reveals VARGG’s advantage in generating structurally clear and biologically meaningful visualizations. Figure 3c shows that VARGG’s Uniform Manifold Approximation and Projection (UMAP) [32] visualization presents distinct cell populations while its Partition-based Graph Abstraction (PAGA) [33] graph preserves essential topological connections between cellular groups. In contrast, other methods exhibit various limitations: SEDR, STAGATE, and stLearn display complex connection patterns that potentially obscure critical biological relationships; GraphST and SpaCAE show insufficient connections to fully capture potential associations between cell populations; DeepST demonstrates moderate connectivity but with less defined cluster boundaries. VARGG uniquely balances cluster definition with inter-cluster connectivity, enabling more accurate identification of cell state transitions and functional relationships in the tumor ecosystem.

Notably, we detected high expression levels of VEGFA [34] and CD74 [35] genes (Fig. 3d, f) and performed GO enrichment. Our experiments showed that CD74 activated the ERK1/2-MAPK signaling pathway by binding to MIF, promoting the proliferation and survival of tumor cells. CD74 also forms a complex with CXC chemokine receptors (such as CXCR2 and CXCR4) and participates in the recruitment of inflammatory and atherosclerotic cells. Elevated VEGFA levels in GBM are associated with increased tumor vascularization, which contributes to tumor growth and resistance to treatment. This mechanism has also been verified in other studies, further confirming its potential as a therapeutic target. In addition, we used volcano plots to show differential gene expression in domains 1, 2, 3, 8, and 10 (|log fold change| ≥ 2, *P* < .05, Fig. 3e, g), and detected high expression of genes such as SPP1, IGFBP5, EMP1, and THY1. Compared with normal tissues, SPP1 is abundantly expressed in GBM, which regulates the expression of PSMA (prostate-specific membrane antigen) through the transcription factor HIF1α and promotes angiogenesis in GBM. Interestingly, IGFBP5 exhibits a dual role in glioblastoma: on the one hand, it promotes tumor cell invasion by promoting epithelial–mesenchymal transition (EMT); on the other hand, it inhibits tumor cell proliferation by inhibiting the Akt signaling pathway. These results are consistent with previous studies.

Importantly, our analysis also identified differential expression of TGDS (TDP-glucose 4,6-dehydratase), extending beyond the established gene signatures associated with glioblastoma progression and immune microenvironment. The functional role of TGDS in human glioblastoma pathophysiology remains unexplored, presenting a novel candidate for further mechanistic investigation in GBM biology.

In summary, our study shows the important application prospects of the VARGG model in glioblastoma research. VARGG accurately reveals the spatial organization of tumors and identifies key genes related to tumor progression and immune microenvironment (VEGFA, CD74, SPP1, IGFBP5, TIMP2, EMP1, THY1). These findings provide an important basis for future glioblastoma treatment strategies and a deeper understanding of disease biology.

### Analysis of molecular heterogeneity and key regulatory pathways in the breast cancer microenvironment ecological niche

**Figure 4:**
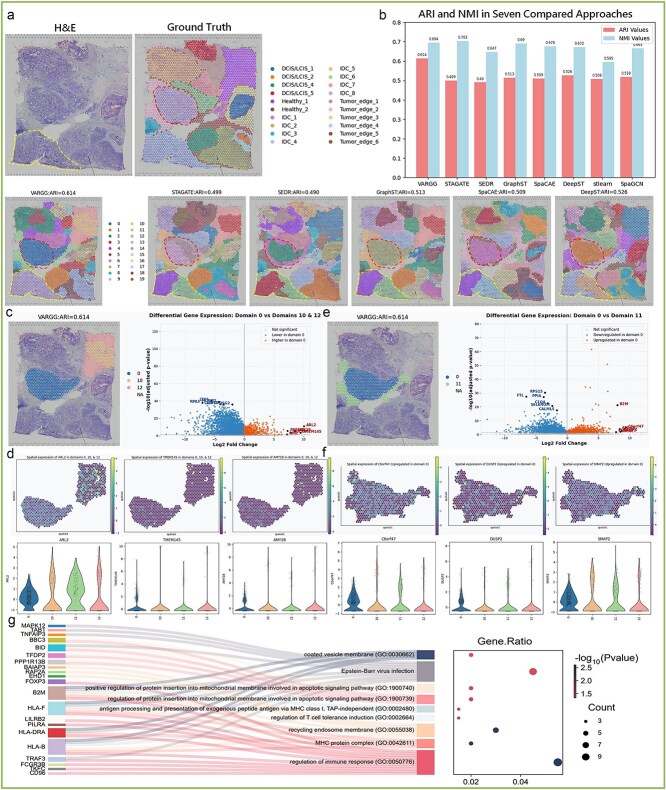
Performance and gene expression analysis in breast cancer tissue spatial transcriptomics: (**a**) H&E-stained section (left) with pathologist annotations (Ground Truth, right) showing invasive ductal carcinoma (IDC), ductal carcinoma *in situ* (DCIS), lobular carcinoma *in situ* (LCIS), tumor–stroma interface, and adjacent normal tissue, with comparative segmentation results from six algorithms below. (**b**) Quantitative performance assessment of spatial domain identification algorithms using Adjusted Rand Index (ARI) and Normalized Mutual Information (NMI) metrics. (**c**) Differential gene expression between IDC (domain 0) and normal tissue (domains 10, 12). Left: VARGG spatial segmentation. Right: volcano plot depicting differentially expressed genes. Top differentially expressed genes (adjusted *P* < .05) are labeled. (**d**) Spatial expression profiles of representative genes (ARL2, TMEM145, AMY2B) in IDC versus normal tissue. Upper panel: spatial visualization; lower panel: violin plots of expression distribution across domains 0, 10, 11, and 12. (**e**) Differential gene expression between tumor core (domain 0) and tumor–stroma interface (domain 11). Left: VARGG segmentation. Right: volcano plot showing significantly altered genes. (**f**) Spatial expression patterns of C6orf47, DUSP2, and SMAP2 comparing tumor core and tumor–stroma interface regions. Upper panel: spatial distribution; lower panel: violin plots quantifying domain-specific expression. (**g**) Sankey diagram illustrating pathway enrichment analysis of 200 differentially expressed genes (100 from IDC versus normal tissue comparison; 100 from tumor core versus tumor–stroma interface comparison; Wilcoxon rank-sum test, adjusted *P* < .05).

This research utilized VARGG to analyze the complex tumor microenvironment (TME) of breast cancer, with results shown in Fig. 4a. The left panel displays H&E staining of breast cancer tissue, the right panel presents ground truth annotations by pathologists, and the lower panel demonstrates the segmentation effects of different algorithms on these microdomains. In a systematic comparison, we comprehensively evaluated the performance of seven spatial domain segmentation algorithms, with results from five algorithms shown in the main figure, while segmentation results from stLearn and SpaGCN are presented in Fig. S3. Notably, VARGG delineated different microenvironment niches and could precisely and completely identify the IDC_4 region and the IDC_8 region (areas circled by yellow and red dashed circles in the figure), whereas the other six models incorrectly segmented this critical IDC region into two different categories. Such erroneous segmentation may lead to misjudgment of tumor heterogeneity, subsequently affecting downstream molecular characterization analysis. Although stLearn identified the IDC_4 region as a single area to some extent, its boundary definition was unclear with numerous scattered confusions, reducing the reliability of precise microenvironment delineation. We quantitatively compared the performance of seven spatial domain segmentation methods in TME characterization (Fig. 4b). VARGG performed best in terms of ARI (0.614) and was second only to the optimal algorithm in Normalized Mutual Information (NMI) [36] (0.684 versus 0.694). However, considering that ARI is more directly related to pathologically validated biological domains, VARGG’s comprehensive performance surpassed other algorithms. Our results strongly support VARGG’s advantage in delineating functionally distinct microenvironment niches in the breast cancer ecosystem.

Based on VARGG’s accurate segmentation of TME, we conducted differential expression analysis between IDC core microenvironment (domain 0) and healthy tissue microenvironments (domains 10 and 12) (Fig. 4c), revealing microenvironment-specific gene expression patterns. In the volcano plot, blue dots represent genes highly expressed in healthy tissue but downregulated in IDC, while orange dots represent genes specifically upregulated in the IDC microenvironment, highlighting molecular reprogramming occurring within malignant niches. To validate key microenvironment-specific genes identified by differential expression analysis, we performed spatial distribution visualization analysis on the most significantly differentially expressed candidate genes. The top six genes specifically upregulated in IDC and the top six genes highly expressed in healthy tissue all showed clear microenvironment-dependent expression patterns. Figure 4d presents the spatial distribution characteristics of IDC upregulated markers ARL2 [37], TMEM145 [38], and AMY2B [39] in key microenvironment domains (domains 0, 10, and 12) (remaining genes shown in Fig. S3). The spatial expression maps and corresponding quantitative analysis precisely depict the expression gradients of these genes between tumor and healthy tissues, providing intuitive evidence for the correlation between gene expression and microenvironment characteristics.

Notably, we observed that ARL2 expression in the IDC core microenvironment (domain 0) was significantly lower than in healthy tissue microenvironments (domains 10 and 12) and the tumor–stroma interface (domain 11). This finding aligns with previous research indicating that low ARL2 expression is associated with enhanced cellular invasiveness, while high ARL2 expression inhibits invasive behavior. TMEM145 exhibits significantly high methylation status in all stages (I–III) of breast invasive ductal carcinoma, accompanied by upregulated gene expression, making it a potential epigenetic biomarker for breast cancer that may play a key role in cancer progression. Interestingly, although previous studies reported reduced AMY2B expression in various tumors, it has not been thoroughly explored in breast cancer. Our data show that AMY2B exhibits a low expression pattern in the breast cancer microenvironment, providing new insights into the potential role of the amylase family in breast cancer development.

To investigate molecular communication networks between different regions within tumors, we systematically compared transcriptome differences between the IDC core (domain 0) and the tumor–stroma interface (domain 11) (Fig. 4f and Fig. S3). Differential expression analysis revealed significant molecular heterogeneity between the invasive front and tumor core. Notably, VARGG successfully identified fine transition domains with unique gene expression characteristics at the tumor–stroma interface (domain 11) and IDC tissue transition regions. We observed that the C6orf47 gene showed a significant gradient expression pattern in these transition domains. Although the function of C6orf47 in tumor biology has not been fully elucidated, our spatial transcriptome analysis suggests that its expression changes may participate in breast cancer development. Additionally, our observed predominant expression of DUSP2 [40] in the tumor core is consistent with previous research findings that decreased DUSP2 expression levels are closely related to tumor growth. This spatially specific gene expression pattern reflects the unique molecular programs activated by tumors at the invasive front, providing a molecular basis for dynamic interactions between tumor cells and surrounding stromal components.

We conducted systematic functional enrichment analysis on the top 100 most differentially expressed genes from two comparative analyses (IDC versus healthy tissue and IDC versus tumor margin regions), totaling 200 genes (Fig. 4g). The Sankey diagram clearly illustrates the complex association network between differentially expressed genes and enriched biological pathways. Among them, the three most significantly enriched key pathways were regulation of immune response [41], Epstein–Barr virus (EBV) infection [42], and recycling endosome membrane-related pathways [43]. These three pathways all play important roles in the development of IDC. The immune response regulation pathway is characterized by upregulation of key molecules such as programmed death-ligand 1 (PD-L1) and cytotoxic T-lymphocyte-associated protein 4 (CTLA-4), causing imbalanced distribution of T-cell subpopulations and forming a specific immune microenvironment. Activation of the EBV infection pathway is consistent with the high EBV positivity rate reported in previous studies of IDC samples, with research showing that EBV infection is significantly associated with poor prognostic factors such as hormone receptor negativity and high histological grade. Abnormalities in the recycling endosome membrane pathway lead to sustained activation of receptor tyrosine kinase signaling and integrin cycling disorders, promoting enhanced tumor cell migration and invasion capabilities. Notably, there are potential interactions among these three pathways, such as recycling endosome-mediated PD-L1 membrane transport regulating immune evasion processes, suggesting they may synergistically promote IDC progression. These findings provide a new theoretical foundation for developing integrative therapeutic strategies targeting invasive breast cancer.

### The gene expression atlas of VARGG in the hippocampal region and its application in the pathology of Alzheimer’s disease

**Figure 5:**
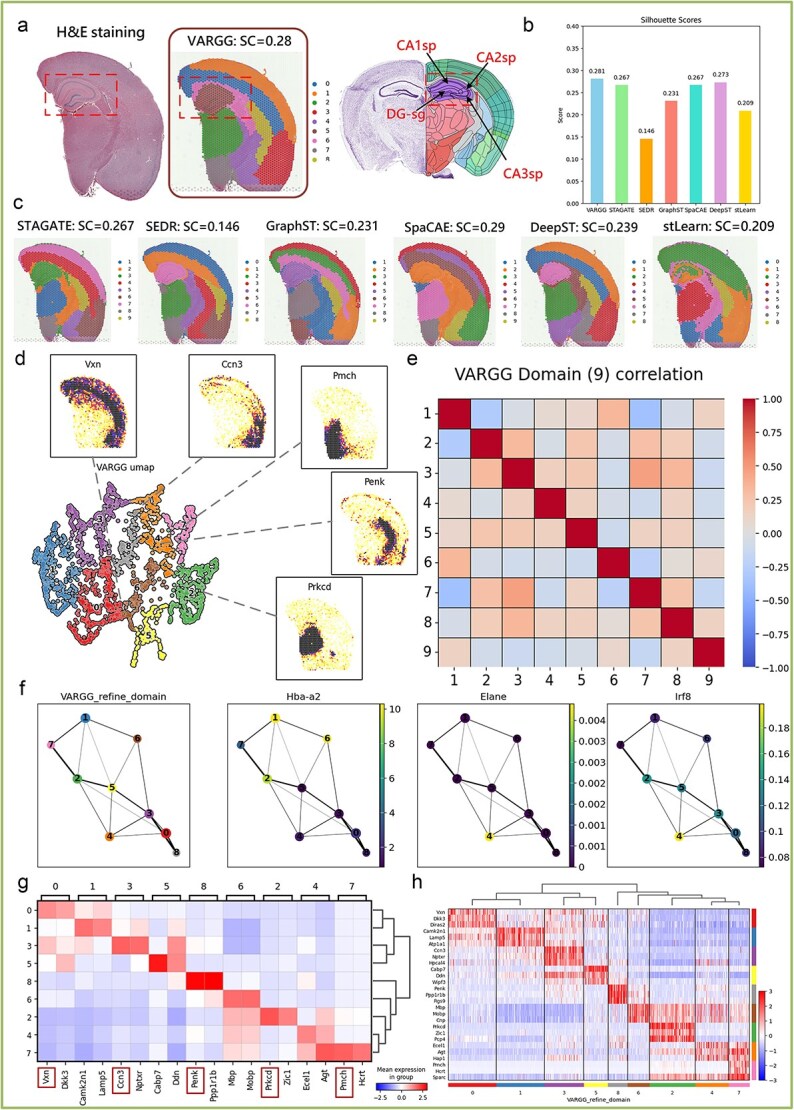
Comprehensive spatial clustering and gene expression analysis in adult mouse brain tissue. (**a**) Left: H&E staining image of FFPE adult mouse brain tissue section. Right: the hippocampus identified by VARGG is clearer. (**b**) Comparison of SC scores for spatial domain identification of models. (**c**) Comparison of hippocampal region segmentation across different algorithms. (**d**) UMAP projection showing spatial gene expression distribution, with insets highlighting specific gene expression patterns. (**e**) Correlation matrix between the nine clusters defined by VARGG, showing gene expression relationships. (**f**) PAGA network representation of gene relationships based on VARGG clustering, depicting potential pathways and interactions. (**g**) Heatmap displaying average expression levels of selected marker genes in VARGG-identified clusters, with intensity indicating expression magnitude. (**h**) Hierarchical clustering heatmap of differential gene expression patterns in spatial domains defined by VARGG in FFPE mouse brain tissue, with expression values normalized as *Z*-scores.

We analyzed formalin-fixed, paraffin-embedded (FFPE) adult mouse brain [44] tissue sections to study key hippocampal subregions: CA1, CA2, CA3, and the dentate gyrus (DG-sg [45]) (Fig. 5a). The hippocampus is crucial for memory formation and retrieval, and diseases like Alzheimer’s often first affect this area. As shown in Fig. 5b and 5c, the VARGG model (SC = 0.281) demonstrated higher clustering consistency with the ground truth (GT), indicating its superiority in identifying cell heterogeneity in the hippocampus.

We observed spatial gene expression distribution within the mouse brain through UMAP projection. The five genes depicted in Fig. 5d (Vxn, Ccn3, Pmch, Penk, Prkcd [46]) are closely related to nervous system functions, affecting memory formation, emotional responses, and neurodegenerative disease progression. Visualizing these genes’ expression helps understand their roles in normal brain function and disease states, crucial for hippocampal research and AD [47] treatment. A correlation matrix revealed gene expression relationships among the nine hippocampal areas defined by VARGG, suggesting distinct gene expression differences across these spatial domains (Fig. 5e). We also used a PAGA network to interpret relationships and potential pathways between clusters based on specific genes like Hba-a2, Elane, and Irf8, revealing possible biological pathways and complex interactions within the hippocampus (Fig. 5f). Heatmaps showcased average expression levels of selected marker genes in VARGG-identified clusters, providing dynamic insights into gene expression within different clusters (Fig. 5g). Hierarchical clustering analysis highlighted patterns of differentially expressed genes in the spatial domains defined by VARGG, depicting unique gene expression profiles in each spatial domain of the FFPE mouse brain tissue (Fig. 5h). This result offers a crucial perspective for understanding functional changes in the hippocampus in both healthy and AD pathological states.

Our findings are particularly important for AD research, as the hippocampus is one of the primary brain regions affected early in the disease. By deeply analyzing gene expression patterns in the hippocampus, we can identify genes and pathways critical to AD progression. These discoveries provide new insights into the molecular basis of AD and potential therapeutic strategies targeting the disease.

### Spatial gene expression analysis of mouse embryo development on the Stereo-seq platform

**Figure 6:**
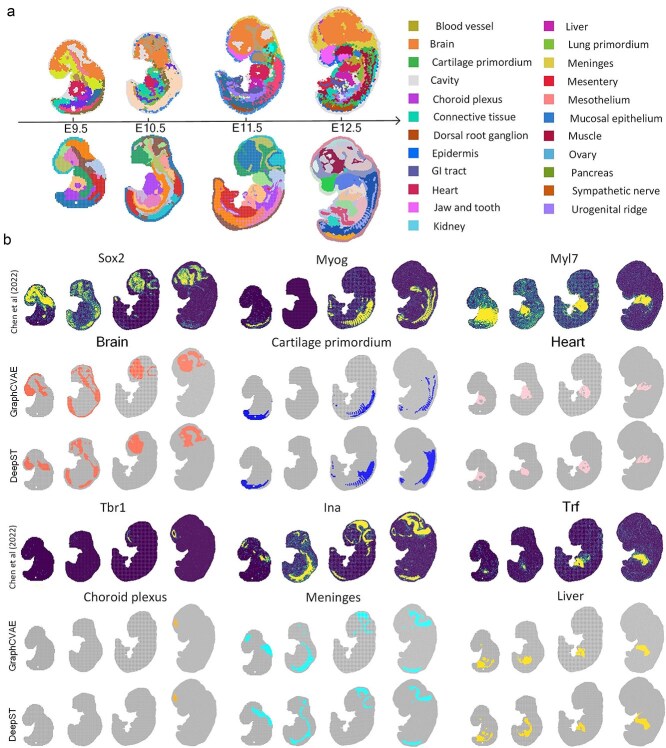
Spatiotemporal gene expression patterns in mouse embryo development on the Stereo-seq platform. (**a**) Continuous spatial maps showing mouse embryos at different stages, with the various differentiated tissues marked in the legend on the right. (**b**) Display of high-resolution spatial gene expression profiles of selected marker genes along the developmental timeline (modules VARGG and DeepST). The spatial distribution of each marker gene within the embryo is shown *in situ*, highlighting the specificity of gene expression patterns at different stages of development.

We used the VARGG model to analyze spatial gene expression patterns during mouse embryonic development, revealing the dynamic changes and complexity in early embryonic tissue and organ development. The VARGG model identified continuous closed meninges and specific tissue structures that accounted for <5% at later stages, such as the hypothalamus and olfactory bulb (Fig. 6a). Neurogenesis in the hypothalamus begins at E11.5 and peaks at E12.5, while olfactory bulb development begins at E10.5 and can only be observed at the macroscopic level at E12.5. These findings indicate that the VARGG model is able to integrate ST slice data from different stages, facilitate comparative analysis between stages, and capture the heterogeneity of embryonic development.

We analyzed high-resolution spatial gene expression profiles of selected marker genes along the developmental timeline (Fig. 6b). Compared with the DeepST model, the VARGG model showed finer details in spatial distribution. For example, the VARGG model provided a detailed and continuous expression pattern of Sox2 [48] in the brain, while the DeepST model missed some local expressions. The predictions of the VARGG model were more consistent with the actual data, especially in complex pattern regions such as the heart (Myl7) and cartilage primordium (Myog [49]). VARGG also has a finer description of the expression boundaries of the choroid plexus (Tbr1) and cartilage primordium (Myog). In Fig. 6b, the VARGG model is notably better than DeepST in spatial accuracy and detail for all marker genes. Our experiments show that the VARGG model is better at handling complex tissue structures and distinguishing adjacent regions with different gene expression profiles.

### The generalizability of the VARGG model: applications across multiple spatial transcriptomics platforms

We used the mouse olfactory bulb [50] Slide-seqV2 dataset from the Stereo-seq platform to show the spatial gene expression in different anatomical regions of the olfactory bulb (Fig. S4A). The experimental results show that VARGG can accurately interpret known tissues, has high spatial resolution, and can measure the expression of specific regions in detail, revealing the heterogeneity within the olfactory bulb. The gene expression heat map and the clustered UMAP on the right further emphasize the efficiency of the model in multidimensional data integration. We used violin plots to describe the expression levels of genes in specific regions of the olfactory bulb (Fig. S4B). The expression profile of each gene provides spatial expression information for understanding the complexity of the gene regulatory network in the olfactory bulb.

The high-resolution analysis results of the Slide-seqV2 dataset are shown in Fig. S4C. The spatial distribution of known genes is shown on the left, and the color depth indicates the number of genes in different positions. The middle part uses the stLearn model to identify 8 expression domains, and the right side shows the 14 complete domains identified by the VARGG model.

To further demonstrate VARGG’s capability across multiple platforms, we applied our model to MERFISH datasets of mouse hypothalamic preoptic sections at Bregma−0.09 mm (Fig. S5A) and Bregma−0.04 mm (Fig. S5B). These datasets profile 5557 and 5488 cells, respectively, each with 155 genes. As shown in the leftmost plots, the datasets contain various cell types including astrocytes, multiple endothelial subtypes (Endothelial 1–3), ependymal cells, excitatory and inhibitory neurons, microglia, oligodendrocyte lineage cells at different maturation stages (OD Immature 1–2, OD Mature 1–4), and pericytes.

VARGG model achieved ARI scores of 0.290 and 0.343 on the Bregma−0.09 mm and Bregma−0.04 mm sections, respectively, showing improved performance compared to methods such as STAGATE (ARI = 0.027, 0.036), SEDR (ARI = 0.096, 0.061), GraphST (ARI = 0.076, 0.058), SpaCAE (ARI = 0.146, 0.164), DeepST (ARI = 0.247, 0.259), stLearn (ARI = 0.293, 0.334), and SpaGCN (ARI = 0.240, 0.335). Visual comparison of results demonstrates that our model effectively captures the spatial architecture of the hypothalamic preoptic region, particularly around the third ventricle (the central structure in both sections). The 14 spatial domains identified by VARGG (labeled 0–13) reflect the known cell-type distribution patterns, indicating the model’s potential for preserving both biological structure and cellular heterogeneity of the tissue.

By successfully applying our model across Stereo-seq, Slide-seqV2, and MERFISH platforms, VARGG demonstrates remarkable generalization ability across spatial transcriptomics technologies with varying detection methods, spatial resolutions, and gene coverage. It consistently shows advantages in the accuracy of subdividing spatial domains and can identify complex spatial expression patterns regardless of platform-specific technical differences.

### Key mechanism ablation verification study

Experimental results show that the VARGG model has competitive advantages over other methods. To highlight the advantages of Gaussian noise and multi-head attention mechanism, as well as the impact of ViT, we conducted ablation experiments and designed four VARGG variants. The first variant does not contain Gaussian noise (NG), the second does not use multi-head attention mechanism (NM), the third uses neither multi-head attention mechanism nor Gaussian noise (NGM), and the fourth does not use ViT component (NV). These experiments were conducted on multiple datasets with ground truth labels, including DLPFC, human breast cancer, and MERFISH datasets Bregma0.09 and Bregma0.04. Figure S6 shows that the full VARGG model (including Gaussian noise, multi-head attention mechanism, and ViT) achieves higher ARI values than other variants. Variants lacking multi-head attention mechanism, Gaussian noise (NGM), or ViT (NV) perform poorly, confirming that the simultaneous use of Gaussian noise, multi-head attention mechanism, and ViT can improve the performance of the VARGG model.

## Discussion

The VARGG framework represents a significant advancement in spatial transcriptomics analysis by integrating ViT with variational graph autoencoders, enabling precise identification of spatial domains across diverse biological contexts. By addressing three critical challenges in current methodologies—batch effect correction, dynamic fusion of multimodal data, and adaptive spatial neighborhood construction—VARGG demonstrates robust generalizability across platforms (10x Visium, Slide-seqV2, Stereo-seq, MERFISH) and datasets (human glioblastoma, breast cancer, mouse brain, embryonic development). Its ability to harmonize global contextual information from histology with local gene expression patterns offers unprecedented insights into tissue architecture and cellular heterogeneity.

A key innovation of VARGG lies in its hybrid architecture. The pretrained ViT captures morphological features and global tissue context through self-attention mechanisms, while the graph neural network autoencoder models spatial relationships at multiple scales. This synergy allows VARGG to resolve fine-grained spatial domains even in tissues with highly intermixed cell types, as evidenced by its superior performance in the DLPFC dataset (median ARI = 0.586) and glioblastoma analysis (SC = 0.27). The incorporation of Gaussian noise and multi-layer gated residual networks further enhances model robustness, mitigating overfitting and improving generalizability across datasets—advantages validated through systematic ablation studies.

Compared to existing methods, VARGG addresses longstanding limitations in spatial transcriptomics analysis. While tools like SpaGCN and STAGATE rely on heuristic spatial smoothing or predefined neighborhoods, VARGG dynamically integrates spatial, transcriptional, and morphological data through adaptive graph construction. This capability is critical for capturing multi-scale biological interactions, as shown in its accurate delineation of tumor–stroma interfaces in breast cancer and hippocampal subregions in Alzheimer’s disease models. Furthermore, VARGG outperforms contrastive learning–based approaches (e.g. GraphST) in preserving both cluster separation and inter-domain connectivity, a balance essential for modeling cellular state transitions in complex microenvironments.

The practical implications of VARGG extend across biomedical research. In oncology, its identification of spatially resolved therapeutic targets—such as VEGFA and CD74 in glioblastoma, or ARL2 and TMEM145 in breast cancer—highlights its potential to uncover microenvironment-specific drivers of disease progression. In neuroscience, VARGG’s mapping of gene expression gradients in the hippocampus provides a foundation for exploring early molecular events in neurodegenerative disorders. Additionally, its application to embryonic development datasets demonstrates unique strengths in resolving dynamic spatial patterns, such as the spatiotemporal regulation of Sox2 and Myog during organogenesis.

Despite these advances, VARGG has limitations. The computational complexity of ViT and graph autoencoders may pose challenges for ultra-large datasets (e.g. Stereo-seq and MERFISH with millions of cells). While Gaussian noise improves robustness, optimal noise levels require dataset-specific tuning. Future work could explore lightweight ViT variants or spatial subsampling strategies to enhance scalability. Additionally, expanding VARGG to integrate single-cell resolution data or temporal spatial transcriptomics could further elucidate cellular dynamics in development and disease.

In conclusion, VARGG establishes a versatile framework for spatial transcriptomics analysis. By enabling precise spatial domain identification and microenvironment characterization, it accelerates discoveries in developmental biology, oncology, and neuroscience. The model’s open-source availability ensures broad applicability, inviting further refinements to meet evolving challenges in spatial omics research.

## Methods

### Data description

We integrated multiple high-throughput spatial expression datasets to deepen our understanding of gene expression and spatial distribution in various biological contexts. These include the DLPFC dataset and datasets of human glioblastoma, human breast cancer, and adult mouse brain, providing valuable comparisons and insights into cancer biology, neurodevelopment, and neurodegenerative processes. We also studied mouse embryo datasets, using data from the Stereo-seq and Slide-seqV2 platforms, to investigate mouse embryonic development and understand early developmental stages and cell differentiation mechanisms. Additionally, we included the MERFISH dataset, which contains two anterior hypothalamic sections of mouse selected at Bregma−0.04 mm and Bregma−0.09 mm. In Supplementary Table 1, we list in detail the number of spots and the number of genes in some datasets.

### Data preprocessing

The data processing method used by the VARGG model not only uses gene expression information, but also integrates spatial location and morphological characteristics. This integration is crucial to enhance the overall processing method. The following are the specific methods of these calculations:

1. Spatial weight calculation

Spatial weights ${W}_s$ are crucial for quantifying the proximity of data points within tissue samples in spatial transcriptomics. For any two data points $i$ and $j$, the spatial weight is defined as follows:


(1) \begin{equation*} {W}_s\left[i,j\right]=\left\{\begin{array}{ll}1& \mathrm{if}\ j\ \mathrm{is}\ \mathrm{one}\ \mathrm{of}\ \mathrm{the}\ \mathrm{k}\hbox{-}\mathrm{nearest}\ \mathrm{neighbors}\ \mathrm{of}\ i\\{}0& \mathrm{otherwise}\end{array}\right. \end{equation*}

2) Gene expression weight calculation

Gene expression weights ${W}_g$ are determined to understand the correlations and functional relationships between genes in spatial transcriptomics data. For gene expression profiles ${X}_i$ and ${X}_j$ within the data matrix $X$, the gene expression weights are calculated as follows:


(2) \begin{equation*} {W}_g\left[i,j\right]=1-\frac{X_i\cdot{X}_j}{\Vert{X}_i \Vert \Vert{X}_j \Vert } \end{equation*}

This calculation employs the cosine similarity between gene expression vectors, transformed into a measure of dissimilarity.

3) Morphological similarity calculation

On the 10x Visium platform, the VARGG model employs morphological similarity calculations using a ViT neural network. The ViT segments tissue images and transforms them into 1000-dimensional latent variables. Principal component analysis (PCA) then reduces these to the top 100 dimensions, focusing on the most informative features. Morphological similarity between data points is calculated using Euclidean distance on these PCA-reduced features. This calculation is crucial for enhancing data processing accuracy and relevance on the 10x Visium platform.

For data from other platforms, morphological similarity is not used during the spatial data augmentation phase. The VARGG model adapts to different platforms by customizing the use of morphological similarity based on data characteristics and availability. This flexibility ensures the model’s effective operation under various conditions, maximizing the potential of the data used.

The morphological similarity between data points is calculated using the Euclidean distance formula on these PCA-reduced features. For two data points with PCA features ${X}_{pca,i}$ and ${X}_{pca,j}$, the similarity *W_m_* is determined as


(3) \begin{equation*} {W}_m\left[i,j\right]=1-\frac{\sqrt{\sum \limits_k{\left({X}_{pca, ik}-{X}_{pca, jk}\right)}^2}}{\max (d)} \end{equation*}

4) Composite weight matrix calculation

For data from the 10x Visium platform, the composite weight matrix ${W}_{all}$ incorporates an integration of spatial weights ${W}_s$, gene expression weights ${W}_g$, and morphological similarity weights ${W}_m$. This matrix is specifically tailored to combine various dimensions of data, offering a comprehensive perspective of spatial transcriptomics unique to the 10x Visium datasets.

It is calculated as follows:


(4) \begin{equation*} {W}_{all}={W}_s\times{W}_g\times{W}_m \end{equation*}

For data from other platforms where morphological similarity weights ${W}_m$ are not applied, the composite weight matrix is adjusted accordingly:


(5) \begin{equation*} {W}_{all}={W}_s\times{W}_g \end{equation*}

This adjustment ensures that the composite weight matrix remains relevant and accurate for datasets where morphological data may not be available or applicable.

5) Spatial data augmentation

The spatial data augmentation process enhances the gene expression profile of each data point by considering the influence of its spatial neighbors. This enhancement is achieved by adding the contribution of neighboring gene expressions, weighted by the composite weight matrix ${W}_{all}$, to the original gene expression data:


(6) \begin{equation*} {X}_i^{\prime }={X}_i+\alpha \sum \limits_j{W}_{all}\left[i,j\right]{X}_j,{X}_i^{\prime}\in{R}^{N\times O} \end{equation*}

In the data preprocessing section, we adjusted the adjacent weight factor $\alpha$ and explained its impact. Adjusting $\alpha$ moderates the contributions of neighboring gene expressions to the augmented profile. A lower $\alpha$ value reduces neighbor influence, potentially underutilizing spatial information, while a higher $\alpha$ value might overly emphasize neighbor influence, obscuring the original data. Therefore, finding an appropriate $\alpha$ value is crucial for balancing true expression with spatial information utilization.

To demonstrate the impact of different $\alpha$ values, we included a box plot in Fig. S7, showing the effects on the DLPFC dataset. This plot visually illustrates how different $\alpha$ settings affect the results, aiding in parameter adjustment based on experimental data.

### Dimension reduction and multi-head attention

After spatial data augmentation, we apply PCA to reduce the dimensionality of the enhanced gene expression data.

The multi-head attention mechanism, with two embedded linear layers, adjusts input dimensions before computing dot products between queries and keys to derive attention scores. This enables efficient mapping of input features to a suitable space and flexible adjustment of feature dimensions. The multi-head attention mechanism accommodates diverse input dimensions, ensuring effective processing at different scales and demonstrating strong adaptability to diverse inputs, making it a key component of the model architecture.

In our model implementation, the number of attention heads is chosen based on systematic studies of model performance. We use 8 attention heads for the input layer, 4 attention heads after the first encoder layer, and 8 attention heads for the decoder layer. This design allows the model to focus on different levels of feature representation at different processing stages. The 8 heads in the input layer comprehensively capture complex relationships in the original data; the 4 heads in the intermediate encoding layer reduce the number of parameters, preventing overfitting; while the 8 heads in the decoder layer enhance the accuracy of feature reconstruction. Notably, these head number settings can be adjusted according to the characteristics of different datasets to adapt to data structures of varying complexity.

For each layer, we ensure that the embedding dimension is divisible by the number of heads to evenly distribute the feature space. For example, when the first encoder layer dimension is 64, 4 heads allow each head to process a 16-dimensional subspace; when the decoder layer is 64, 8 heads allow each head to process an 8-dimensional subspace. Our implementation also removes the bias term in the attention calculation, a design based on our experimental findings that bias-free design exhibits better generalization performance on spatial transcriptomics data.

This mechanism allows the model to focus on different representational subspaces simultaneously, providing a comprehensive understanding of the data’s contextual relationships. This is particularly advantageous for capturing complex patterns in high-dimensional data.

Mathematically, the multi-head attention mechanism is formulated as follows:


(7) \begin{equation*} \mathrm{MultiHead}\left(Q,K,V\right)=\mathrm{Concat}\left({\mathrm{head}}_1,{\mathrm{head}}_2,\dots, {\mathrm{head}}_h\right){W}^O \end{equation*}

(8) \begin{equation*} {\mathrm{head}}_i=\mathrm{Attention}\left(Q{W}_i^Q,K{W}_i^K,V{W}_i^V\right) \end{equation*}

(9) \begin{equation*} \mathrm{Attention}\left(Q,K,V\right)=\mathrm{softmax}\left(\frac{Q{K}^T}{\sqrt{d_k}}\right)V \end{equation*}

In these equations, $Q,K$ and $V$ represent the query, key, and value matrices. ${W}^Q,{W}^K$, and ${W}^V$ are weight matrices for the *i*-th head’s query, key, and value. ${W}^O$ is the output weight matrix. ${d}_k$ is the dimension of the key vectors.

For the data-enhanced gene expression data ${X}^{\prime }$, the dimensions will not change after being processed by the multi-head attention mechanism. Additionally, experiments were conducted on the DLPFC dataset to evaluate different dimensional reductions’ impact on the multi-head attention mechanism, focusing on the ARI values (as shown in Fig. 2c). These experiments highlight the importance of optimally balancing dimensionality reduction with model performance.

### Graph construction

In VARGG, the graph is constructed by developing an adjacency matrix, where each element represents the spatial relationship between data points. A value of “1” in the matrix signifies neighboring points, while “0” indicates no direct connection between points. This matrix is primarily constructed using the KDTree method, known for its efficiency in processing spatial data. Moreover, VARGG offers flexibility in graph construction techniques by providing alternatives like BallTree [51] and Euclidean methods to accommodate different data characteristics, enhancing the model’s applicability and efficiency across various datasets.

### Denoising autoencoder

In the denoising autoencoder designed for gene expression data, the encoder applies PyTorch’s linear transformations [52] to process the pre-handled gene expression matrix $X$ of dimensions *N* × *M*.


(10) \begin{equation*} \mathrm{Encoder}(X)={Z}_E,X\in{R}^{N\times M},{Z}_E\in{R}^{N\times G} \end{equation*}

where $N$ is the number of spots, $M$ is the dimension after PCA dimensionality reduction, $G$ is the dimension of the last layer of the linear layer, and ${Z}_E$ is the output of the encoder.

The decoder then reconstructs the output by combining the encoder’s output with the subsequent variational autoencoder’s output. This reconstruction aims to match the dimensions of the original input $X$, ensuring a faithful representation of the data.


(11) \begin{equation*} \mathrm{Decoder}\left(Z+{Z}_E\right)={X}^{\prime },{X}^{\prime}\in{R}^{N\times M},Z\in{R}^{N\times R} \end{equation*}

Among them, $Z$ is the output of the variational autoencoder, $R$ is the dimension of its output, ${X}^{\hbox{'}}$ is the output of the decoder, and the meaning of other letters remains unchanged.

The model employs the mean squared error (MSE) loss function to measure the fidelity of reconstruction, optimizing to minimize the difference between the reconstructed output and the original input.


(12) \begin{equation*} {L}_{mse}=\frac{1}{N}\sum \limits_{i=1}^N{\left({\hat{X}}_i-{X}_i\right)}^2 \end{equation*}

This approach effectively captures the essential features of the gene expression data while mitigating noise, making the model robust for further analysis.

### Variational autoencoder

In the variational autoencoder (VAE) designed for processing gene expression data, the encoding phase incorporates Gaussian noise to enhance the model’s robustness against the variability inherent in biological datasets.


(13) \begin{equation*} {H}^{(0)}=\mathrm{Gaussian}\left({Z}_E\right),{H}^{(0)}\in{R}^{N\times G} \end{equation*}

where ${H}^{(0)}$ is the data of $Z{}_E$ after Gaussian noise processing, and is also the input of the first layer of the variational autoencoder.

The architecture employs a series of RGGCN layers to create a compact feature representation, and the process can be summarized as follows:

The lower-dimensional feature matrix after noise enhancement is obtained through the RGGCN layers:


(14) \begin{equation*} {H}^{\left(l+1\right)}=\mathrm{ELU}\left(\mathrm{RGGCN}\left({H}^{(l)},A\right)\right) \end{equation*}

where ${H}^{(l)}$ and ${H}^{\left(l+1\right)}$ are the input and output of the *l*th graph convolutional layer. $A$ is the adjacency matrix implemented in the above graph construction, activation function uses ELU.

The mean $\mu$ and log-variance $\log \left({\sigma}^2\right)$ of the latent space are then derived from the final feature matrix:


(15) \begin{equation*} \mu =\mathrm{RGGCN}\left({H}^{(L)},A\right) \end{equation*}

(16) \begin{equation*} \log \left({\sigma}^2\right)=\mathrm{RGGCN}\left({H}^{(L)},A\right) \end{equation*}

In the reparameterization step, we utilize specific parameters to generate the latent variable $Z$ according to the modified equation (refer to equation 17 in the manuscript). The random variable $\theta$, sampled from a standard normal distribution, ensures that the VAE captures the stochastic nature of gene expression.


(17) \begin{equation*} Z=\mu +\log \left({\sigma}^2\right)\ast \theta, Z\in{R}^{N\times R} \end{equation*}

Following the calculation of $Z$, the decoder reconstructs the inner product of $Z$ to form the transformed adjacency matrix, providing a representation of the data’s relational structure (refer to equation 18 in the manuscript).


(18) \begin{equation*} p\left(A|Z\right)=\mathrm{sigmoid}\left(Z{Z}^T\right) \end{equation*}

Our optimization framework incorporates a comprehensive loss function that extends beyond standard VAE components to enhance the transformed adjacency matrix’s definition and to provide a robust solution for capturing spatial context. The total loss ${L}_{\mathrm{total}}$ is formulated as a combination of three weighted components:


(19) \begin{equation*} {L}_{mse}=\frac{1}{N}\sum \limits_{i=1}^N{\left({\hat{X}}_i-{X}_i\right)}^2 \end{equation*}

(20) \begin{equation*} {L}_{klp}=-\frac{1}{2}\sum \left(1+\log \left({\sigma}^2\right)-{\mu}^2-{\sigma}^2\right) \end{equation*}

(21) \begin{equation*} {L}_{adj}=\mathrm{BinaryCrossEntropy}\left(p\left(A|Z\right),A\right) \end{equation*}

(22) \begin{equation*} {L}_{total}= mw\cdot{L}_{mse}+ kw\cdot{L}_{klp}+ aw\cdot{L}_{adj} \end{equation*}

Here, ${L}_{mse}$ assesses the reconstruction quality, ${L}_{klp}$ imposes a regularization on the latent space, and ${L}_{\mathrm{adj}}$ ensures the accuracy of the reconstructed graph structure. The hyperparameters *mw*, *kw*, and *aw* calibrate the influence of each loss component within the overall optimization process.

Leveraging PyTorch’s built-in Adam optimizer, we efficiently handle sparse gradients and noisy datasets, particularly beneficial for large-scale data and high-dimensional spaces. Its capability for automatic learning rate adjustments is crucial for improving model performance and expediting convergence.

### Cluster analysis and visualization

After obtaining the latent variable *Z*, we perform clustering using the Leiden method to identify spatial domains. To further understand the data’s structure, we use PAGA and UMAP for visualization.

PAGA abstracts the data into a graph, showing the interrelationships between clusters, while UMAP effectively represents high-dimensional data in two or three dimensions.

### Evaluated metrics and criteria

Adjusted Rand Index (ARI) assesses the similarity between the clustering result and the ground truth, adjusting for chance.


(23) \begin{align*} &ARI\notag\\&=\frac{\sum \limits_{ij}\left(\!\begin{array}{c}{n}_{ij}\\{}2\end{array}\right)-\left[\sum \limits_i\left(\begin{array}{c}{n}_{i.}\\{}2\end{array}\right)\sum \limits_j\left(\begin{array}{c}{n}_{.j}\\{}2\end{array}\right)\right]/\left(\begin{array}{c}n\\{}2\end{array}\right)}{\frac{1}{2}\left[\sum \limits_i\left(\begin{array}{c}{n}_{i.}\\{}2\end{array}\right)+\sum \limits_j\left(\begin{array}{c}{n}_{.j}\\{}2\end{array}\right)\right]-\left[\sum \limits_i\left(\begin{array}{c}{n}_{i.}\\{}2\end{array}\right)\sum \limits_j\left(\begin{array}{c}{n}_{.j}\\{}2\end{array}\right)\right]/\left(\begin{array}{c}n\\{}2\end{array}\right)} \end{align*}

Here, ${n}_{ij}$ is the count of spots in both cluster $i$ and cluster $j$, ${n}_{i.}$ and ${n}_{.j}$ are the counts of spots in cluster $i$ and cluster $j$, respectively, and $n$ is the total number of spots.

Normalized Mutual Information (NMI) is a statistical method used to compare how similar two different groupings (or clusters) are in a dataset, considering the chance of random matching. It uses Mutual Information (MI) to measure the shared information between these groupings. The MI shows how much knowing one group helps to predict the other. This measure is then adjusted by the average uncertainty (entropy) of both groupings. It is given by


(24) \begin{equation*} MI\left(U,V\right)=\sum \limits_{i=1}^{c_U}\sum \limits_{j=1}^{c_V}P\left(i,j\right)\log \left(\frac{P\left(i,j\right)}{P(i)P(j)}\right) \end{equation*}

Entropy ($H(U)$ for clustering $U$ and $H(V)$ for clustering $V$) measures the uncertainty or randomness of the clustering assignment, calculated as


(25) \begin{equation*} H(U)=-\sum \limits_{i=1}^{c_U}P(i)\log \left(P(i)\right) \end{equation*}

(26) \begin{equation*} H(V)=-\sum \limits_{j=1}^{c_V}P(j)\log \left(P(j)\right) \end{equation*}

Finally, NMI is calculated using these components, yielding a value between 0 (no mutual information) and 1 (perfect correlation):


(27) \begin{equation*} NMI\left(U,V\right)=\frac{2\times MI\left(U,V\right)}{H(U)+H(V)} \end{equation*}

This formula ensures that NMI is normalized and can be used to reliably compare clustering results across different datasets and methods.

In addition to some of the above indicators, we further enhance our evaluation of clustering quality using additional metrics. These include the silhouette coefficient (SC), which assesses the similarity of objects within their own cluster as opposed to other clusters; the DBI, which calculates the average “similarity” between clusters, aiming for lower values for better clustering distinction; and the CH score, which complements our analysis by evaluating the effectiveness of clustering. The SC is determined by intra-cluster distances and the minimum distance to other clusters, while the DBI considers distances between elements and their cluster centroids, as well as the centroids of different clusters.

### From differential expression to GO enrichment

We used the Scanpy library for single-cell RNA [53] sequencing analysis. Differential expression analysis was performed using the limma [54] package. We set a log fold change threshold of 2 to identify highly up- and downregulated genes. Differentially expressed genes were subjected to GO enrichment analysis using the clusterProfiler package (v4.2.2) [55]. During data processing and visualization, we used the Python libraries pandas and matplotlib to calculate *Z*-scores and create bar charts to display GO terms related to the screened genes to ensure that they met specific criteria.

**Key points**

- VARGG combines a pretrained Vision Transformer (ViT) with graph neural networks to enhance global and spatial contextual analysis.
- Utilizes multi-layer gated residual graph neural networks and Gaussian noise to improve feature representation and generalizability.
- Demonstrated high accuracy in spatial domain identification across various datasets, including human glioblastoma and mouse embryos.
- Employs techniques like multi-head attention and variational autoencoders for effective handling of complex spatial data.
- Accurately maps spatial domains and cellular heterogeneity, offering insights for identifying key molecular markers and therapeutic targets.

## Supplementary Material

Supplemental_Figures_elaf018

Table_elaf018

Figure_S1_elaf018

Figure_S2_elaf018

Figure_S3_elaf018

Figure_S4_elaf018

Figure_S5_elaf018

Figure_S6_elaf018

Figure_S7_elaf018

Figure_captions_elaf018

## Data Availability

(1) The DLPFC (Dorsolateral Prefrontal Cortex) dataset, accessible within the spatialLIBD package (http://spatial.libd.org/spatialLIBD); (2) data pertaining to glioblastoma, breast cancer, and mouse brain, available on the 10X Genomics website (https://support.10xgenomics.com/spatial-gene-expression/datasets); (3) mouse embryo data, which can be downloaded from the China National GeneBank’s Stomics platform (https://db.cngb.org/stomics/mosta); (4) Slide-seqV2 datasets are available at the Broad Institute Single Cell Portal at https://singlecell.broadinstitute.org/single_cell/study/SCP815/highly-sensitive-spatial-transcriptomics-at-near-cellular-resolution-with-slide-seqv2#study-summary; (5) the processed Stereo-seq data from mouse olfactory bulb tissue are accessible at https://github.com/JinmiaoChenLab/SEDR_analyses; (6) the MERFISH dataset is available from https://github.com/zhengli09/BASS-Analysis. The utilization of these publicly available datasets not only augments the transparency of our research but also contributes to reproducible scientific results. Additionally, to facilitate reproducibility and allow the community to verify and build upon our work, the code used in this study is available on GitHub. The repository includes scripts, usage instructions, and additional resources necessary to replicate our analysis and results. The repository can be accessed at https://github.com/w2260584531/VARGG-main.

## Notes

**Mengqiu Wang** is a graduate student at the Academy of Artificial Intelligence, Beijing Institute of Petrochemical Technology. Her expertise is bioinformatics and machine learning.

**Zhiwei Zhang** is a graduate student at the Academy of Artificial Intelligence, Beijing Institute of Petrochemical Technology. His expertise is bioinformatics and machine learning.

**Lixin Lei** is a graduate student at the Academy of Artificial Intelligence, Beijing Institute of Petrochemical Technology. His expertise is machine learning.

**Kaitai Han** is a graduate student at the Academy of Artificial Intelligence, Beijing Institute of Petrochemical Technology. His expertise is deep learning.

**Zhenghui Wang** is a graduate student at the Academy of Artificial Intelligence, Beijing Institute of Petrochemical Technology. Her expertise is bioinformatics.

**Ruoyan Dai** is a graduate student at the Academy of Artificial Intelligence, Beijing Institute of Petrochemical Technology. Her expertise is bioinformatics.

**Zijun Wang** is a graduate student at the Academy of Artificial Intelligence, Beijing Institute of Petrochemical Technology. His expertise is deep learning.

**Chaojing Shi** is a graduate student at the Academy of Artificial Intelligence, Beijing Institute of Petrochemical Technology. His expertise is deep learning.

**Xudong Zhao** is a graduate student at the Academy of Artificial Intelligence, Beijing Institute of Petrochemical Technology. His expertise is deep learning.

**Qianjin Guo** is a professor at the Academy of Artificial Intelligence, Beijing University of Petrochemical Technology. His main research fields include intelligent equipment and systems, machine learning, biomedical imaging, and ultrafast spectroscopy.
