# Supplementary material for: VARGG: a deep learning framework advancing precise spatial domain identification and cellular heterogeneity analysis in spatial transcriptomics
Source: Brief Funct Genomics. 2025 Nov 23;24:elaf018. doi: 10.1093/bfgp/elaf018 (PMC12640549; doi:10.1093/bfgp/elaf018)
Supplement: Supplemental_Figures_elaf018 [file supplemental_figures_elaf018.docx]

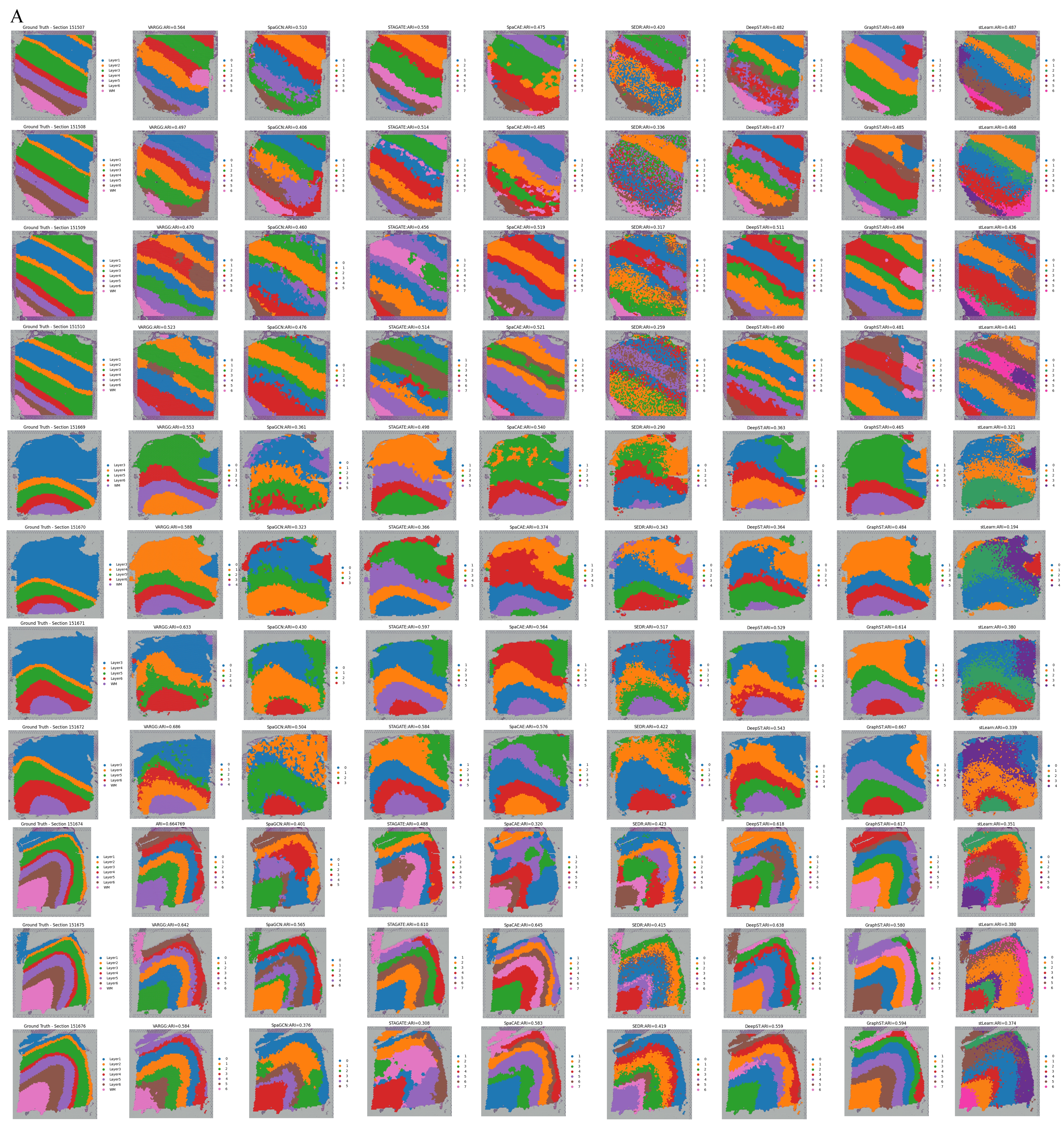


**Figure S1. Performance of different spatial domain detection methods on the DLPFC dataset.**


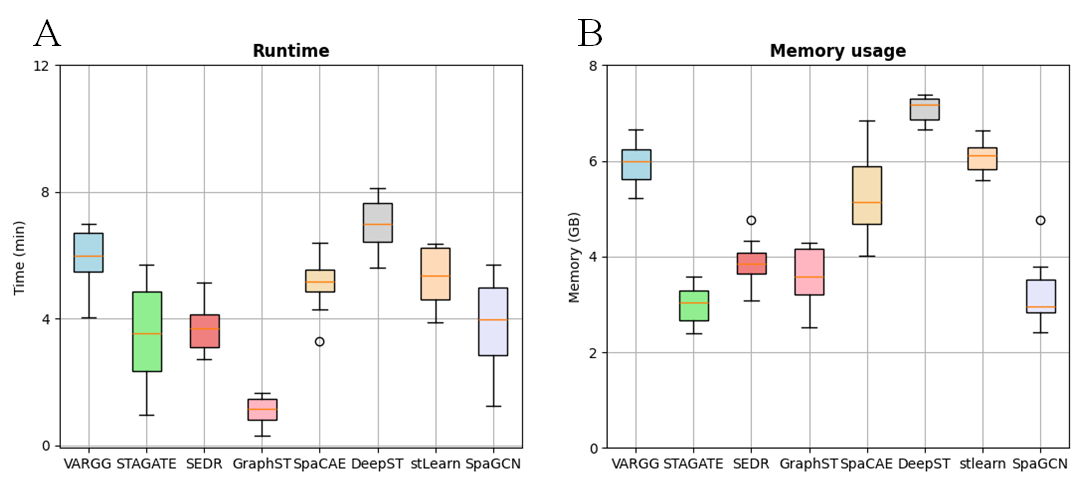


**Figure S2. Comparative Runtime and Memory Usage of Clustering Algorithms on DLPFC Datasets: (A)** Running time (in minutes) of each algorithm applied to 12 slides from the DLPFC dataset, with all models running on the GPU. **(B)** Memory usage (in GB) of each algorithm on the same dataset.

**
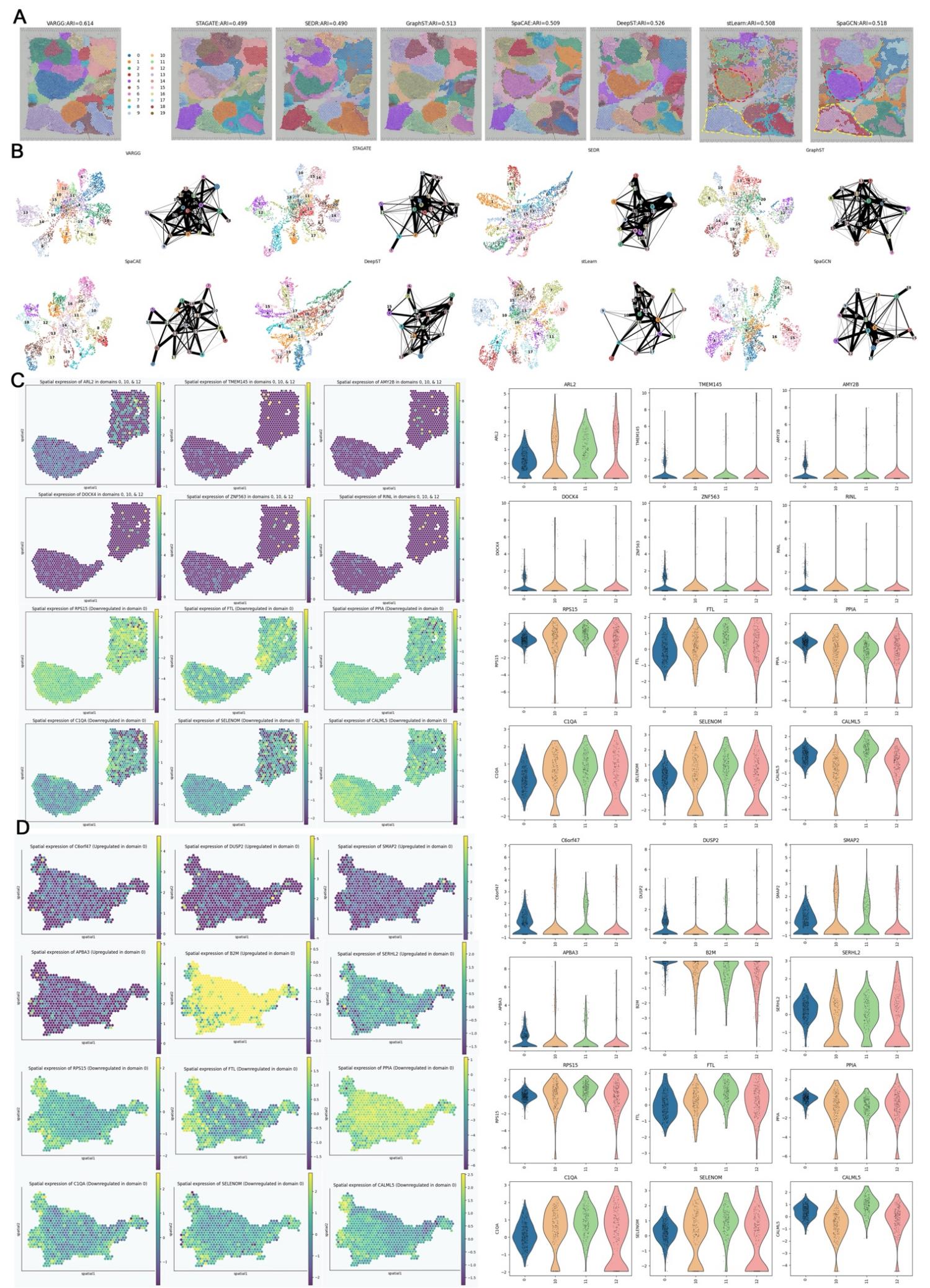
**

**Figure S3. VARGG outperforms existing methods in spatial domain identification and molecular characterization in glioblastoma.** (A). Comparison of spatial domain segmentation results from eight algorithms on a human glioblastoma dataset. Each panel shows domain partitioning by different methods and their corresponding Adjusted Rand Index (ARI) values. VARGG (leftmost) demonstrates superior domain identification capability compared to SEDR, GraphST, SpaCAE, DeepST, stLearn, and SpaGCN. **(B)**. UMAP visualization of spatial domain structures and trajectory analysis across algorithms. The left panels show two-dimensional projections of identified clusters, while the right panels display corresponding trajectory connections between domains. VARGG exhibits clear cluster boundaries and coherent domain relationships. **(C)**. Spatial distribution of differentially expressed genes between IDC core region (domain 0) and healthy tissue regions (domains 10 and 12). The left panel shows spatial distribution maps of 12 differentially expressed genes (6 upregulated and 6 downregulated) across the tissue section, while violin plots on the right quantify expression level differences between domains. **(D)**. Spatial distribution of differentially expressed genes between IDC core region (domain 0) and tumor-stroma interface (domain 11). The maps illustrate the distribution patterns of 12 differentially expressed genes (6 upregulated and 6 downregulated) in the tissue (left panel) and their quantitative expression analysis across different domains (right panel).


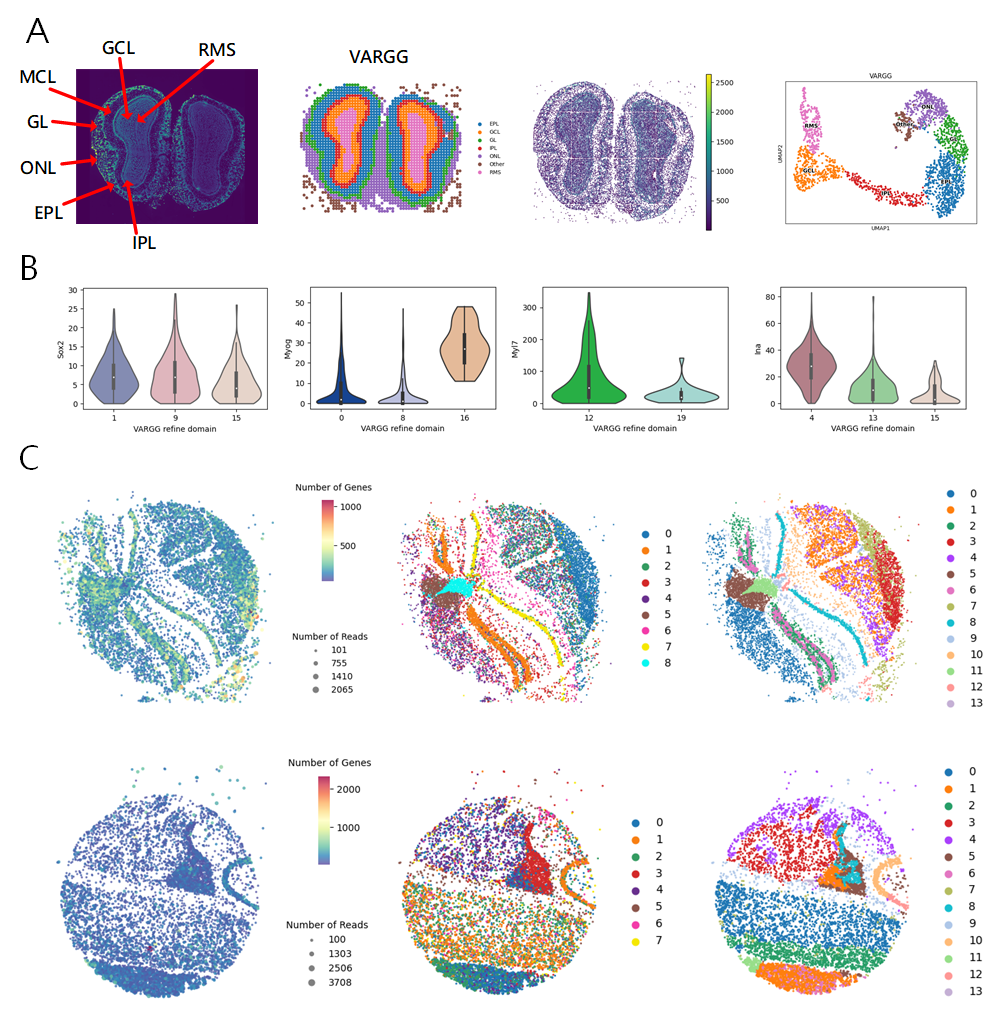


**Figure S4. Comparative Analysis of Spatial Transcriptomics using Stereo-seq and Slide-seqV2 Platforms**:(A) Visualization of spatial gene expression from a mouse olfactory bulb dataset obtained via the Stereo-seq platform. The left panel shows anatomical areas within the olfactory bulb, including the Mitral Cell Layer (MCL), Glomerular Layer (GL), External Plexiform Layer (EPL), highlighted with red lines. The middle panel demonstrates spatial segmentation of gene expression using the VARGG algorithm, while the right panel displays gene expression heatmaps and UMAP clustering.**(B)** Violin plots comparing gene expression metrics within specific regions identified by the refined VARGG algorithm, including gene and transcript counts across identified areas, highlighting expression heterogeneity within the olfactory bulb.**(C)** High-resolution analysis using the Slide-seqV2 dataset. The left panel shows the spatial distribution of known gene numbers at different locations, with color intensity indicating gene quantity. The middle panel presents 8 different expression domains identified using the stLearn model, each represented by a different color. The right panel shows 14 complete domains identified by our VARGG model.


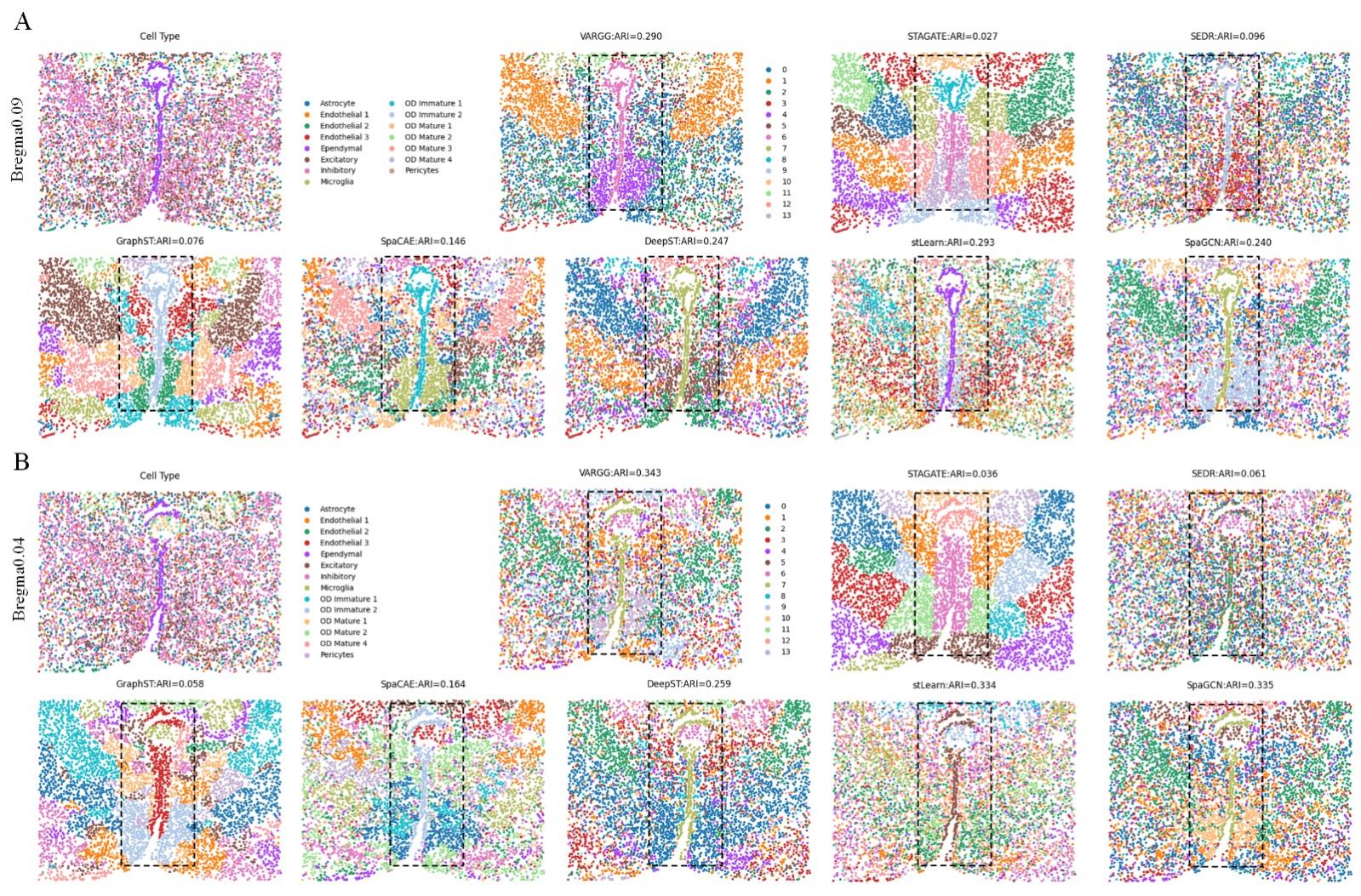


**Figure S5. Spatial domain identification by VARGG and comparison methods on MERFISH mouse hypothalamic datasets: (A)** Visualization of identified spatial domains on the Bregma-0.09 mm section by different methods. The leftmost plot shows ground truth cell types, while other plots display spatial domains identified by VARGG and comparison methods. **(B)** Visualization of identified spatial domains on the Bregma-0.04 mm section by different methods.


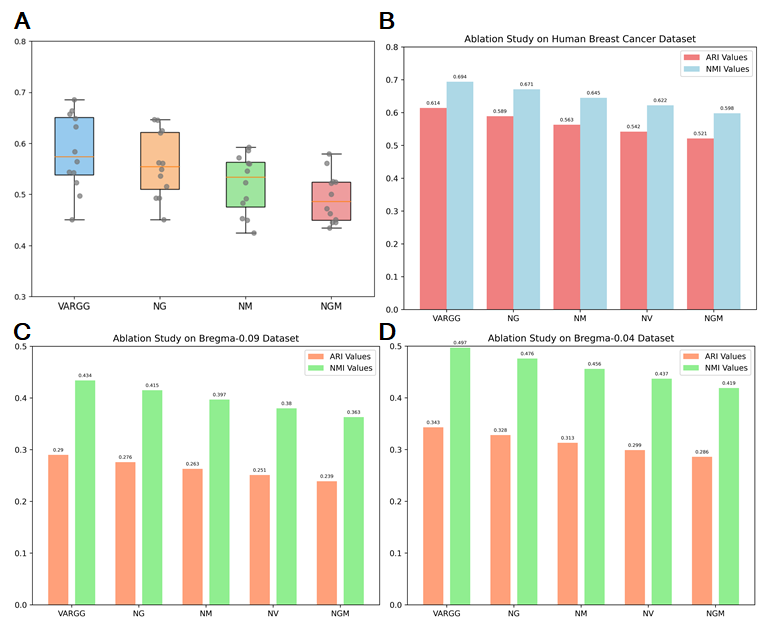


**Figure S6. Ablation Study Results of the VARGG Model on Various Datasets:(A)** Comparison of performance metrics using box plots for VARGG and its variants (NG, NM, NGM) on the DLPFC dataset. **(B)** Comparison of Adjusted Rand Index (ARI) and Normalized Mutual Information (NMI) values for VARGG and its variants on the human breast cancer dataset. **(C)** Comparison of ARI Values and NMI Values on the Bregma-0.09 dataset. **(D)** Comparison of ARI Values and NMI Values on the Bregma-0.04 dataset.


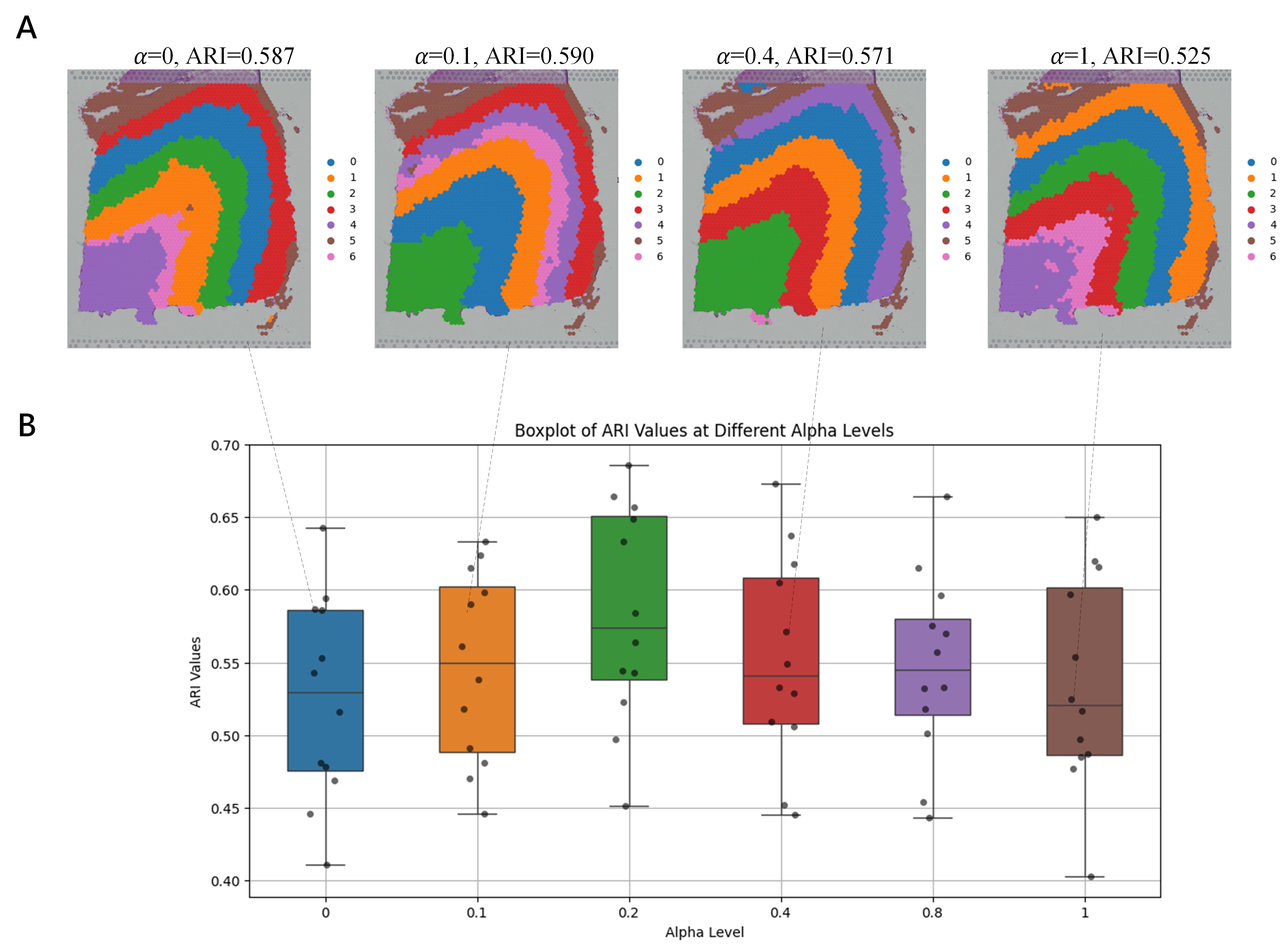


**Figure S7. Impact of Different Alpha Levels on Clustering Outcomes and ARI Values in the DLPFC Dataset: (A)** displays clustering maps for four alpha levels (0.1, 0.4, 0.8, 1.0), showing how varying the adjacent weight factor (𝛼) influences spatial gene expression clustering within the DLPFC dataset. Each map is labeled with the corresponding ARI score, illustrating the quality of clustering at each alpha level. **(B)** presents a box plot of ARI values across different alpha levels, quantifying the statistical variability and performance consistency of clustering as alpha varies, helping to visualize the optimal balance between spatial and gene expression data integration.
