# Supplementary material for: VARGG: a deep learning framework advancing precise spatial domain identification and cellular heterogeneity analysis in spatial transcriptomics
Source: Brief Funct Genomics. 2025 Nov 23;24:elaf018. doi: 10.1093/bfgp/elaf018 (PMC12640549; doi:10.1093/bfgp/elaf018)
Supplement: Table_elaf018 [file table_elaf018.docx]

**Supplementary Table 1 Summary of some of the datasets used in this study**

| **Dataset** | **Slice** | **The number of spots** | **The number of genes** |
| --- | --- | --- | --- |
| DLPFC | 151507 | 4226 | 20494 |
|  | 151508 | 4384 | 20083 |
|  | 151509 | 4634 | 20732 |
|  | 151510 | 3661 | 20475 |
|  | 151669 | 3498 | 20583 |
|  | 151670 | 4110 | 20338 |
|  | 151671 | 4015 | 21037 |
|  | 151672 | 3639 | 10725 |
|  | 151673 | 3673 | 21267 |
|  | 151674 | 3592 | 21897 |
|  | 151675 | 3460 | 20783 |
|  | 151676 | 3798 | 20806 |
| Human Glioblastoma |  | 3468 | 1186 |
| Human Breast cancer | Section_1 | 3789 | 36601 |
| Adult Mouse brain |  | 2264 | 19465 |
| Mouse Embryo | 9.5E1S1 | 5913 | 25568 |
|  | 10.5E1S1 | 18408 | 25201 |
|  | 11.5E1S1 | 30124 | 26854 |
|  | 12.5E1S1 | 51365 | 27810 |
| Stereo-seq | Mouse Olfactory Bulb | 2316 | 27106 |
| Slide-seqV2 | Puck_180413_7 | 12286 | 3235 |
|  | Puck_180819_19 | 9699 | 2555 |
| MERFISH | Bregma0.09 | 5557 | 155 |
|  | Bregma0.04 | 5488 | 155 |
