# Supplementary figures and images for: VARGG: a deep learning framework advancing precise spatial domain identification and cellular heterogeneity analysis in spatial transcriptomics
Source: Brief Funct Genomics. 2025 Nov 23;24:elaf018. doi: 10.1093/bfgp/elaf018 (PMC12640549; doi:10.1093/bfgp/elaf018)

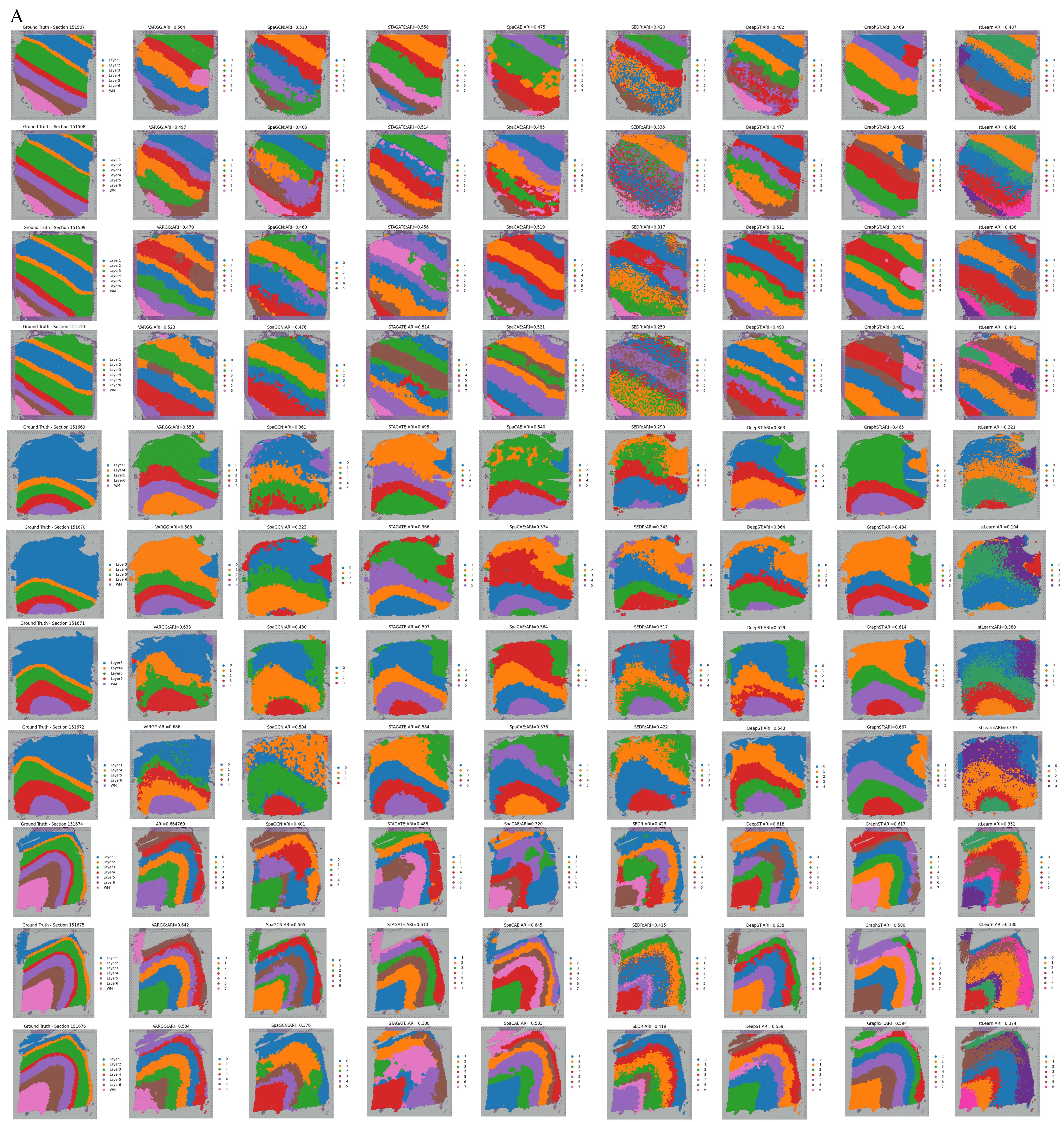

Supplement: Figure_S1_elaf018 [file figure_s1_elaf018.jpeg]

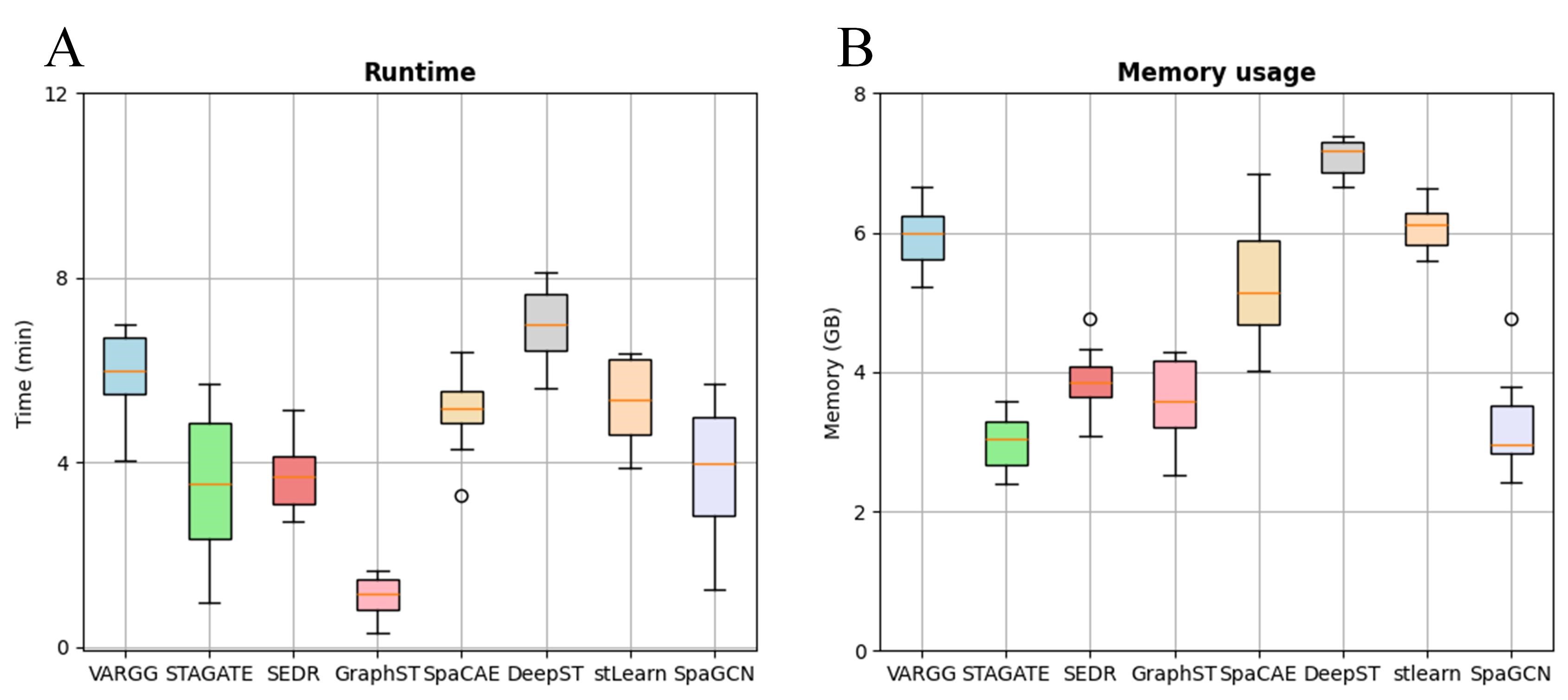

Supplement: Figure_S2_elaf018 [file figure_s2_elaf018.jpeg]

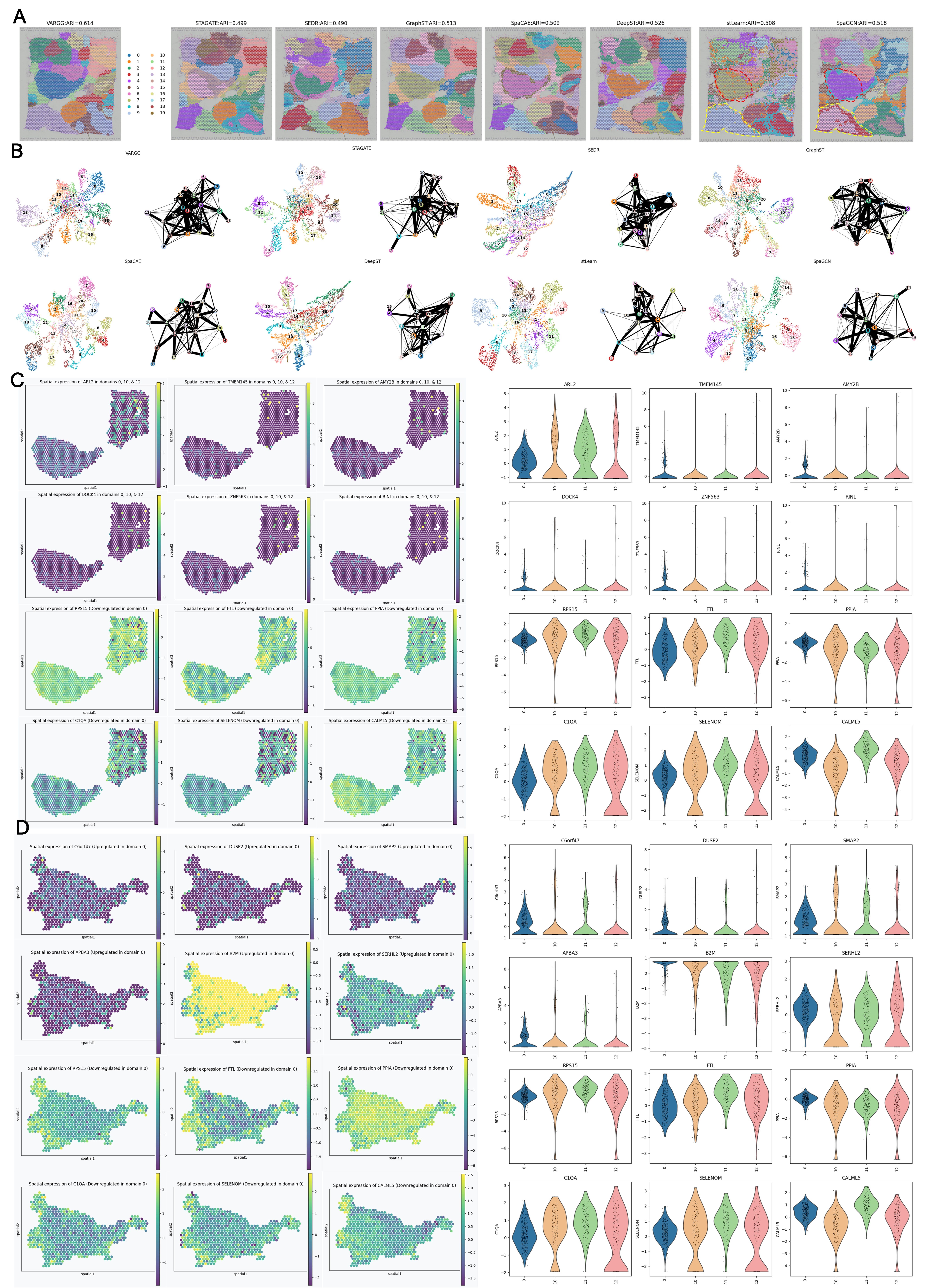

Supplement: Figure_S3_elaf018 [file figure_s3_elaf018.jpeg]

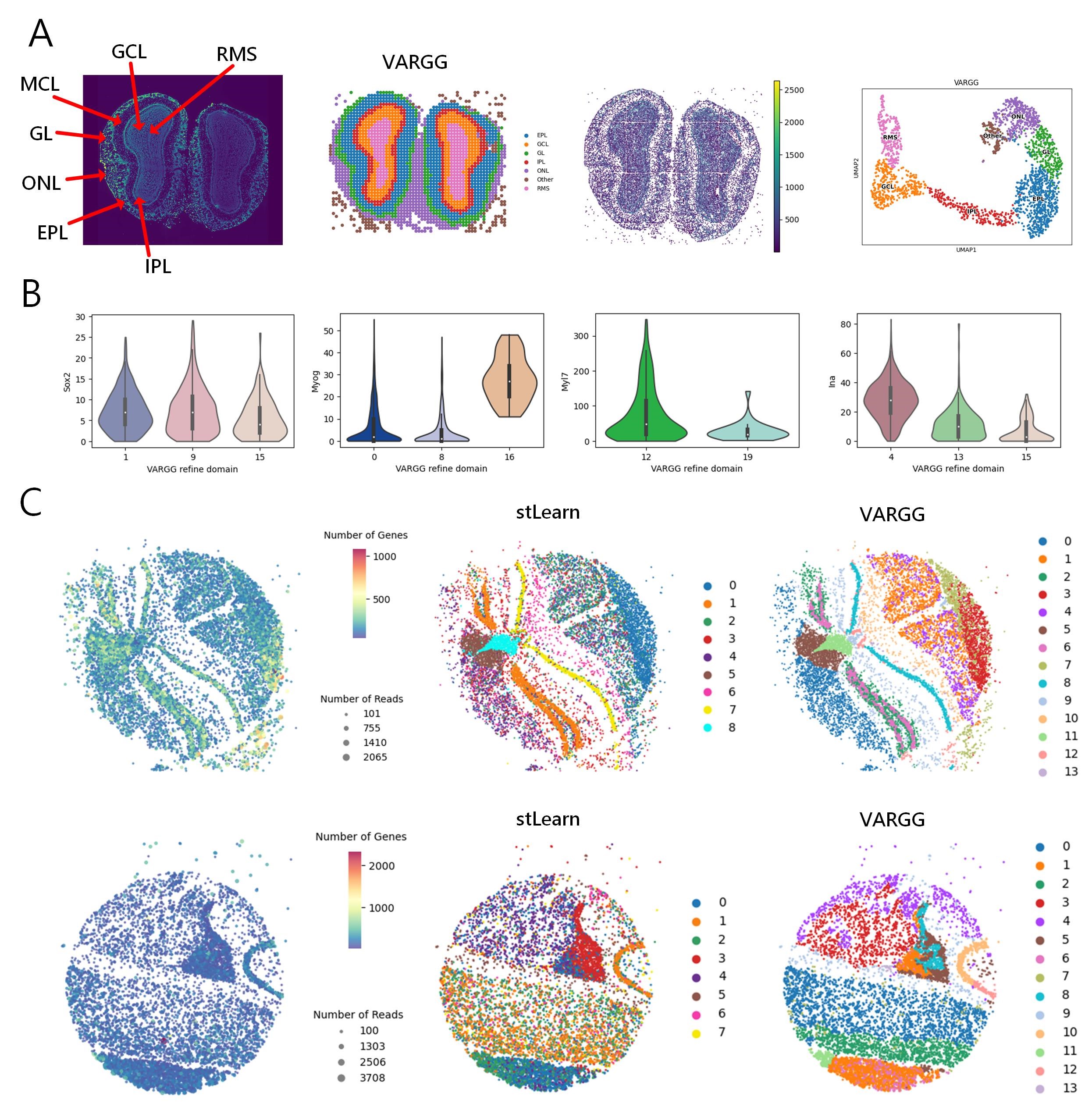

Supplement: Figure_S4_elaf018 [file figure_s4_elaf018.jpeg]

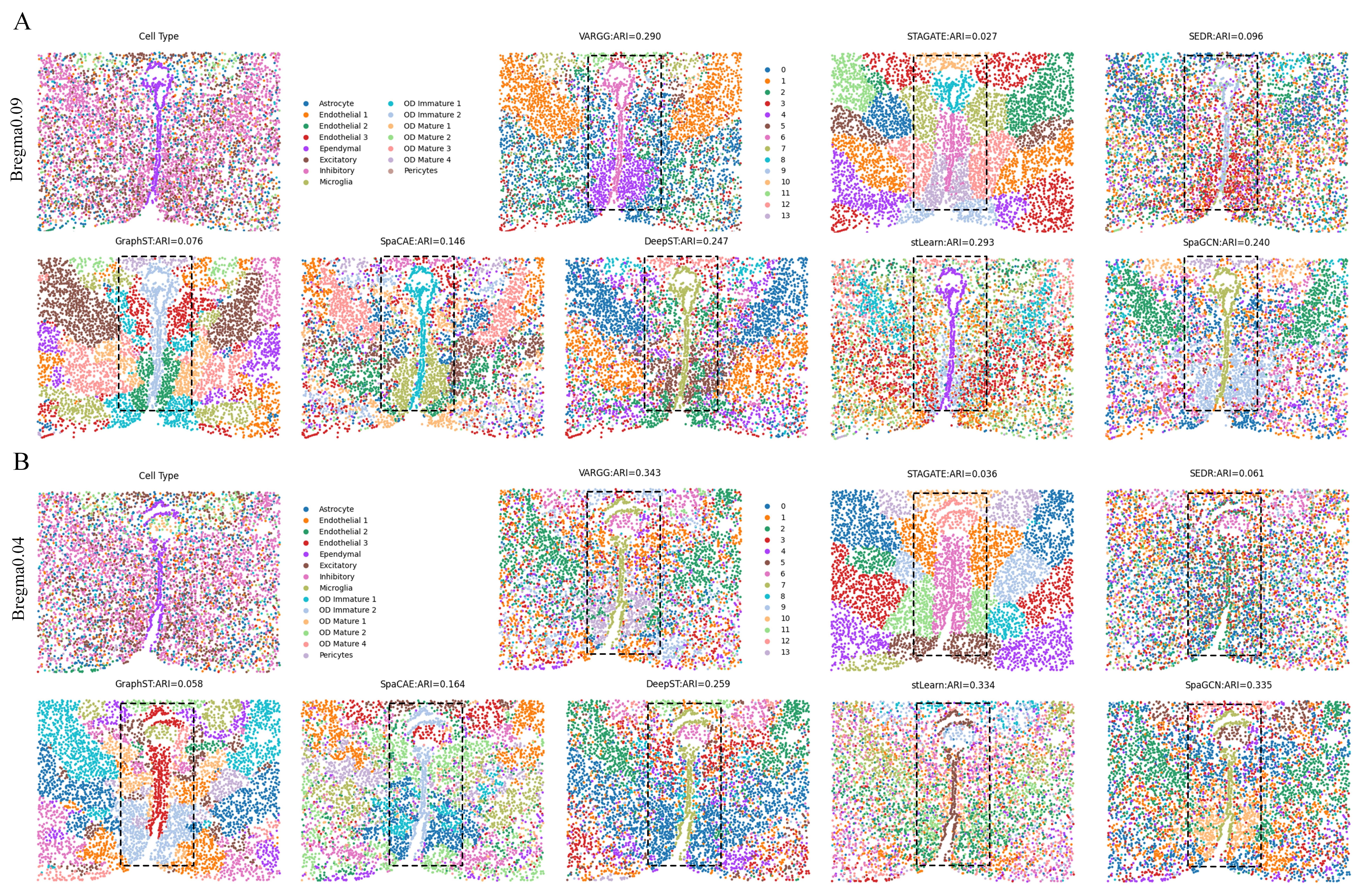

Supplement: Figure_S5_elaf018 [file figure_s5_elaf018.jpeg]

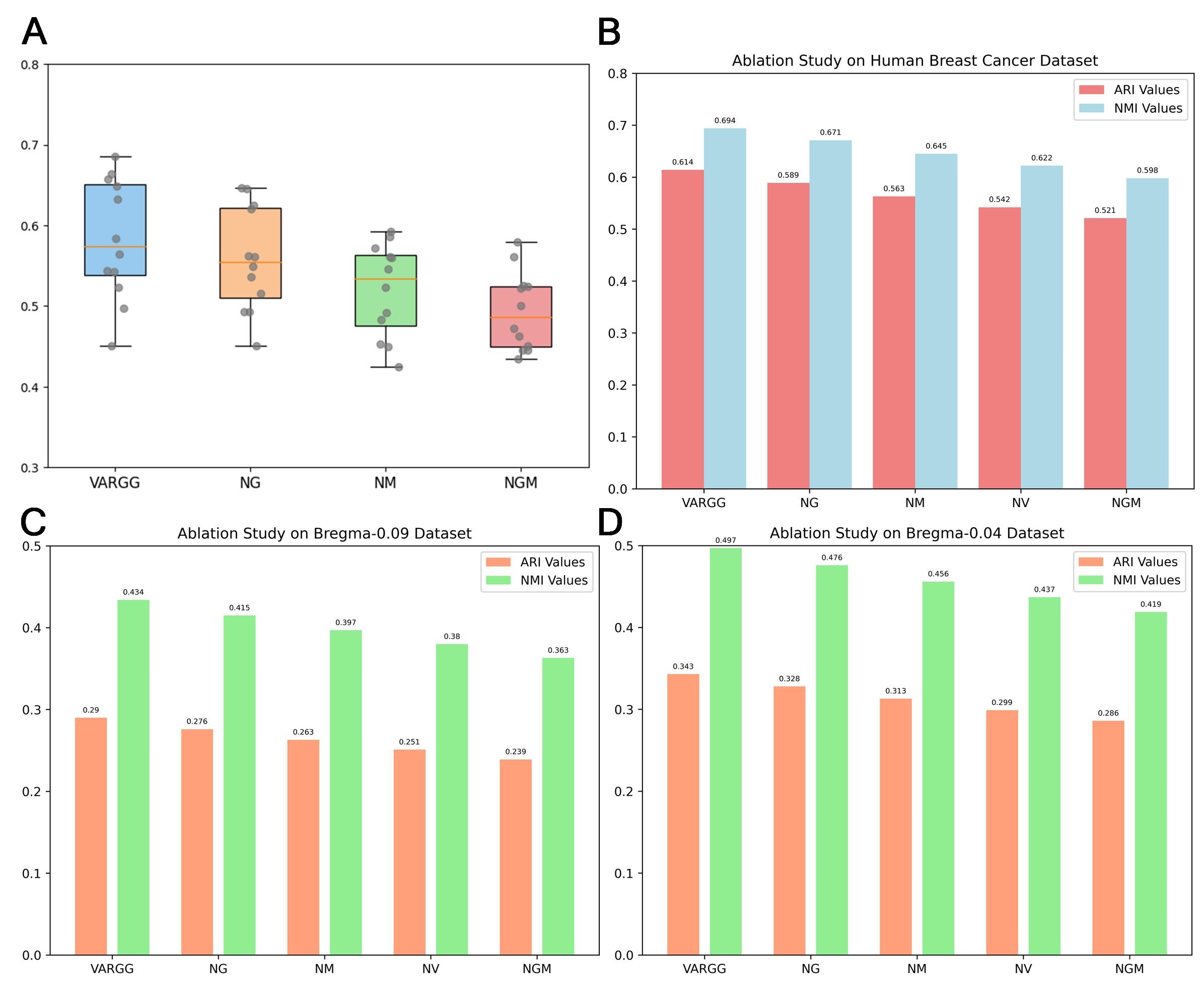

Supplement: Figure_S6_elaf018 [file figure_s6_elaf018.jpeg]

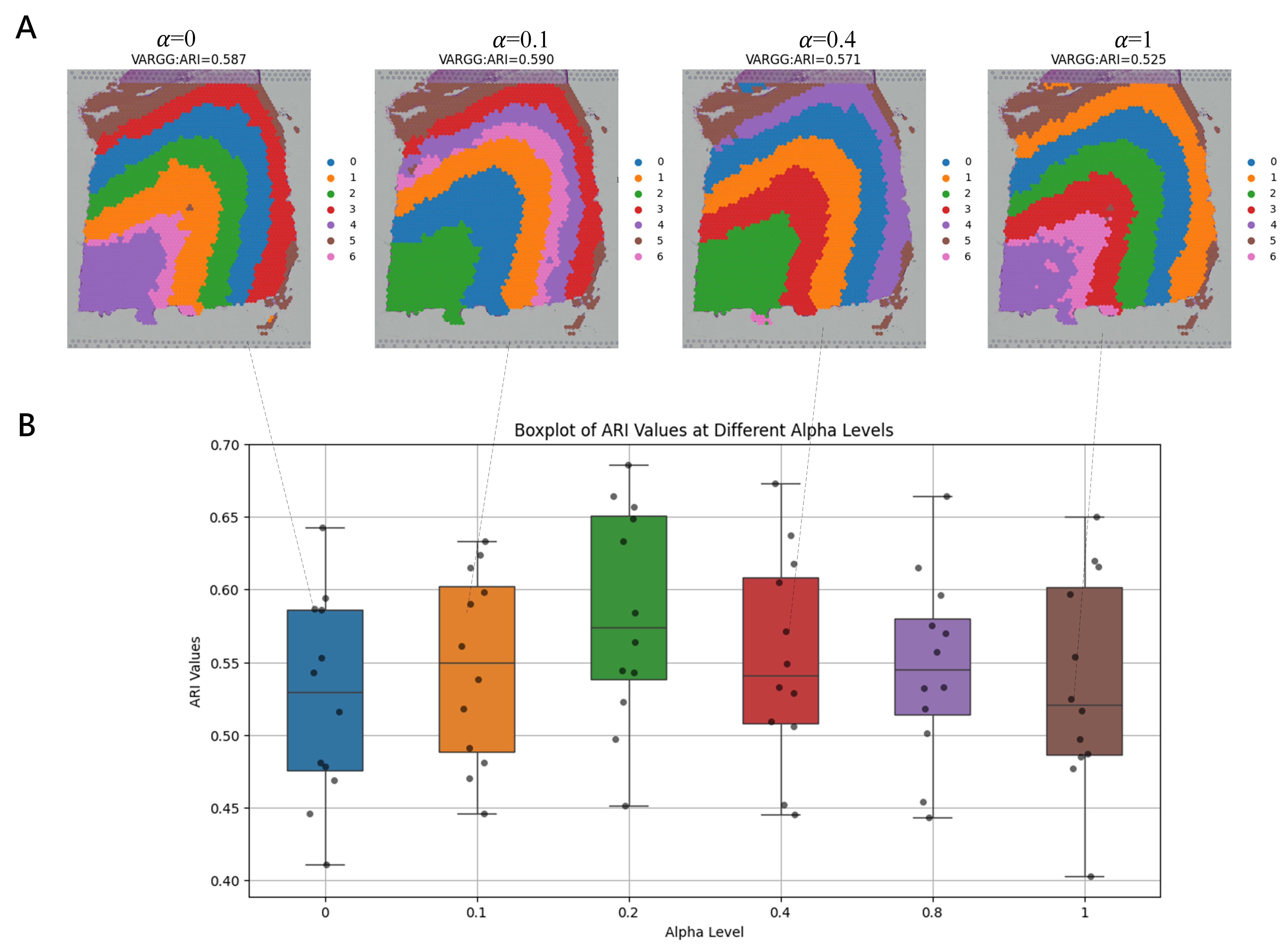

Supplement: Figure_S7_elaf018 [file figure_s7_elaf018.jpeg]
